# Downregulation of EphA2 stability by RNF5 limits its tumor-suppressive function in HER2-negative breast cancers

**DOI:** 10.1038/s41419-023-06188-y

**Published:** 2023-10-10

**Authors:** Xiaojuan Li, Fan Wang, Lu Huang, Mengtian Yang, Ersheng Kuang

**Affiliations:** 1 College of Clinical Medicine, Hubei University of Chinese Medicine, Wuhan, 430061 Hubei China; 2 Zhongshan School of Medicine, Sun Yat-Sen University, Guangzhou, 510080 Guangdong China; 3 Key Laboratory of Tropical Disease Control (Sun Yat-Sen University), Ministry of Education, Guangzhou, 510080 Guangdong China

**Keywords:** Ubiquitylation, Oncogenes

## Abstract

Ephrin receptor A2 (EphA2) plays dual functions in tumorigenesis through ligand-independent tumor promotion or ligand-dependent tumor suppression. However, the regulation of EphA2 tumor-suppressive function remains unclear. Here, we showed that RNF5 interacts with EphA2 and induces its ubiquitination and degradation, decreases the stability and cell surface distribution of EphA2 and alters the balance of its phosphorylation at S897 and Y772. In turn, RNF5 inhibition decreases ERK phosphorylation and increases p53 expression through an increase in the EphA2 level in HER2-negative breast cancer cells. Consequently, RNF5 inhibition increases the adhesion and decreases the migration of HER2-negative breast cancer cells, and RNF5 silencing suppresses the growth of xenograft tumors derived from ER-positive, HER2-negative breast cancer cells with increased EphA2 expression and altered phosphorylation. RNF5 expression is inversely correlated with EphA2 expression in breast cancers, and a high EphA2 level accompanied by a low RNF5 level is related to better survival in patients with ER-positive, HER2-negative breast cancers. These studies revealed that RNF5 negatively regulates EphA2 properties and suppresses its tumor-suppressive function in HER2-negative breast cancers.

## Introduction

Ephrin receptors are a subfamily of cell surface receptor tyrosine kinases (RTKs) that bind to Ephrins as ligands [1, 2]. There are two subgroups of Ephrin receptors, nine type-A members and five type-B members that interact with two subgroups of ligands, four EphrinA and three EphrinB isoforms, to regulate many important physiological processes, including growth, development, differentiation and tumorigenesis. These molecules can exhibit either tumor-promoting functions in a ligand-independent manner or tumor-inhibitory functions in a ligand-dependent manner, consequently regulating unique signal transduction pathways for tumorigenesis [3].

Among these receptors, Ephrin type-A receptor 2 (EphA2) is a prognostic factor in several progressive tumors [4–8]. Inhibition of EphA2 by different genetic or pharmacological approaches has been shown to suppress tumorigenesis [9–12], suggesting that EphA2 is a promising therapeutic target to treat diverse malignant cancers. However, EphA2 also acts as a tumor suppressor under several dysfunctional conditions [13–15]. The ligand-induced dimerization and oligomerization of EphA2 trigger tumor-suppressive signaling, while the EphA2 monomer exhibits pro-tumorigenic activity [16, 17]. In the mechanism of ligand-dependent inhibition and ligand-independent promotion, EphA2 phosphorylation has opposite effects in tumorigenesis: ligand-independent EphA2 Ser897 phosphorylation promotes tumor growth and progression [18–20], while ligand-dependent EphA2 Tyr772 phosphorylation inhibits cell growth, migration and invasion [21, 22]. Furthermore, proteolysis of the ligand-binding domain of EphA2 by metalloproteinases may abolish ligand-dependent inhibition and then induce the tumor-promoting function [23].

The endoplasmic reticulum (ER)-localized 18-kDa RING finger E3 ligase RNF5, also named RMA1, is commonly expressed in tumor cells with the highest expression in breast cancer and melanoma cells and specimens, and the RNF5 expression level is negatively correlated with survival in breast cancer patients [24]. RNF5 regulates several essential processes by targeting different substrates. This molecule controls the stability of STING and MAVS to negatively regulate innate immune responses [25, 26], and two novel substrates of RNF5, ATG4B and S100A8, reveal the important roles of RNF5 in the regulation of antibacterial autophagy and intestinal inflammation [27, 28]; RNF5 is then utilized by viruses for immune evasion [29] and by *Salmonella* for trafficking from endosomes/vacuoles to the cytosol [30]. RNF5 is also linked to ER stress alone or with JAMP to play a role in ER-associated degradation of misfolded proteins [31–34], and RNF5-transgenic mice develop an ER stress-associated muscular disorder [35]. This molecule regulates cell motility by ubiquitinating the focal adhesion protein paxillin [36] and promotes tumor cell resistance to cytoskeletal-targeting anticancer drugs [24]. However, its role in tumorigenesis remains controversial; several studies support the tumor-promoting function of RNF5 in different cancers by targeting multiple substrates [24, 37–39], while a tumor-inhibiting function in breast cancers has also been reported: RNF5 induces the ubiquitination and degradation of glutamine carrier proteins SLC1A5/38A2 to decrease glutamine uptake and suppress the tumorigenesis of breast cancers that carry mutated TP53 [40]. A novel RNF5 inhibitor has been developed recently that significantly rescues F508del-CFTR degradation [41], and the efficacy of RNF5 inhibitors in the treatment of diverse tumors is worthy to be investigated.

Recently, we revealed that RNF5 inhibition suppresses the lytic replication of Kaposi’s sarcoma-associated herpesvirus and the growth of primary effusion lymphoma by targeting EphA3 and EphA4 [42], emphasizing the positive function of RNF5 in tumor virus replication and tumorigenesis. In the present study, we found that RNF5 interacted with EphA2, induced its ubiquitination and degradation, and then RNF5 inhibition increased EphA2 level and cell-surface distribution. As a result, RNF5 inhibition altered the balance of EphA2 phosphorylation at Ser897 and Tyr772, decreased ERK activation and increased p53 expression. Consequently, RNF5 depletion increased cell adhesion and decreased cell migration and then inhibited the growth of xenograft tumors derived from HER2-negative breast cancer cells with increased EphA2 levels and altered EphA2 phosphorylation; these findings identified a novel interplay between RNF5 and EphA2 and a potential therapeutic approach for ER-positive, HER2-negative breast cancers.

## Results

### RNF5 interacts with EphA2 and induces its ubiquitination and degradation

**Fig. 1:**
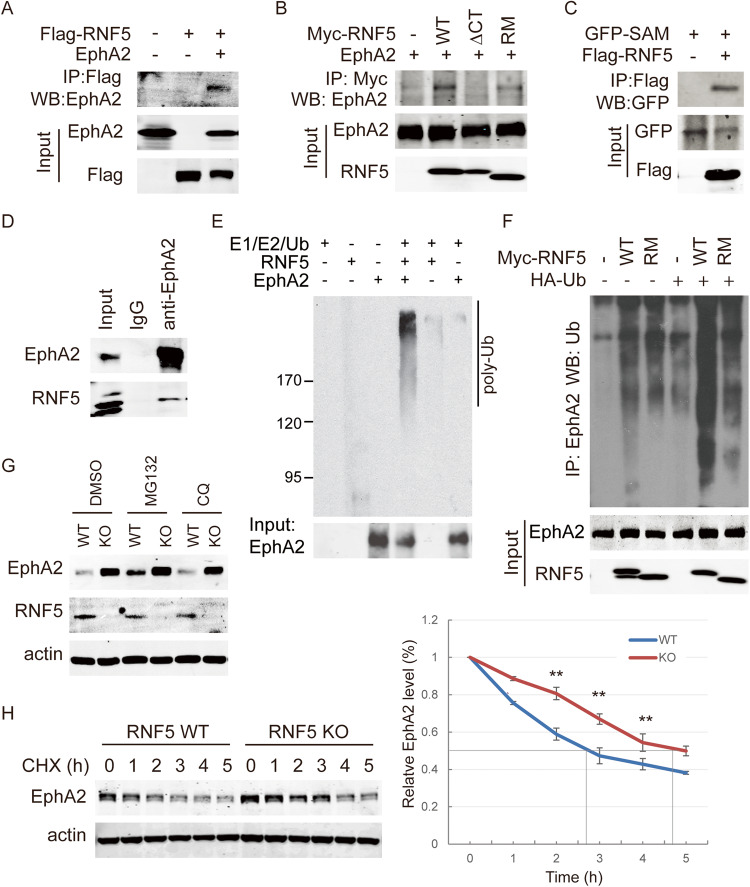
RNF5 interacts with EphA2 and induces its ubiquitination and degradation. **A** The empty vector or Flag-tagged RNF5-expressing plasmid was co-transfected into 293T cells with vector- or EphA2-expressing plasmids. Forty-eight hours post-transfection, the cells were collected, and cell extracts were subjected to immunoprecipitation with anti-Flag antibody. The immunoprecipitated complexes were detected as indicated for the EphA2 interaction. **B** The myc-tagged construct of the wild-type (WT) RNF5, RNF5 with a C-terminal deletion of the transmembrane domain (ΔCT) or RNF5 with a mutation of RING domain (RM) was co-transfected with the EphA2 expression construct, and immunoprecipitation with anti-myc antibody and immunoblotting analysis with anti-EphA2 antibody were performed. **C** GFP-fused EphA2 SAM fragment was expressed in 293T cells with Flag-RNF5 or empty vector for 48 h, and immunoprecipitation with anti-Flag antibody and western blots with anti-GFP antibody were performed as indicated. **D** MCF7 cells were collected and lysed, and immunoprecipitation with IgG control or anti-EphA2 antibody were performed and the complexes were detected by western blots with anti-RNF5 antibody as indicated for their interaction. **E** EphA2 was overexpressed in 293T cells and immunoprecipitated with their antibodies, and these purified proteins conjugated with the beads were subjected to an in vitro ubiquitination reaction with purified RNF5 protein as an E3 ligase. Then, the proteins were pulled down after the reaction and detected by western blots with an anti-ubiquitin antibody. **F** Myc-tagged RNF5 WT or RM construct was co-transfected with an EphA2 construct into 293T cells, and the cells were collected and lysed in lysis buffer containing 1% SDS. After tenfold dilution with lysis buffer to a final concentration of 0.1% SDS, the cell lysates were subjected to immunoprecipitation with the anti-EphA2 antibody and western blots with the anti-Ubiquitin antibody, and input samples were detected by western blots. **G** RNF5 wild-type and RNF5 KO MEFs were left untreated or treated with 10 µg/ml MG132 or 40 µg/ml chloroquine (CQ) for 4 h; the cells were collected, and cell extracts were subjected to western blotting as indicated. **H** RNF5 wild-type and RNF5 KO MEFs were pulse treated with cycloheximide (CHX) (40 µg/ml) for different times. Then, the cells were collected, and cell extracts were detected by western blots as indicated to measure the half-lives of EphA2 in RNF5 WT and KO MEFs. Quantitative measurement of the protein levels was performed based on band grayscale intensity using the LI-COR system and calculated in duplicate experiments; the data are shown as the mean value. **p < 0.01.

By affinity purification and mass spectrometry analysis of RNF5-binding proteins, we identified EphA2 as an RNF5-binding protein. When RNF5 was overexpressed in 293T cells overexpressing EphA2, the ectopic expression of both EphA2 and RNF5 were observed compared with their basal levels and then the assay of immunoprecipitation confirmed that RNF5 interacts with EphA2 (Fig. 1A). The interaction with EphA2 required the C-terminal transmembrane domain of RNF5, while the catalytic activity-dead mutation in the RING domain did not affect the interaction (Fig. 1B), indicating that their interaction required RNF5 membrane-anchoring domain but not E3 ligase activity. EphA2 cytoplasmic region consists of one tyrosine kinase domain, one sterile α motif (SAM) domain and one PDZ domain-binding motif, in which SAM domain and PDZ-binding motif mediate its interactions with cytoplasmic proteins. The SAM domain of EphA2 was able to interact with RNF5 (Fig. 1C), indicated that the SAM domain of EphA2 might be mainly responsible for their interaction. Immunoprecipitation with anti-EphA2 antibody confirmed endogenous RNF5-EphA2 interaction existed in MCF7 cells (Fig. 1D). These results suggest that RNF5 interacts with EphA2.

Next, we investigated whether RNF5 induces the ubiquitination and degradation of EphA2. Immunoprecipitated EphA2 protein from 293T cells overexpressing this protein was incubated with purified RNF5 protein in an in vitro ubiquitination reaction. After the reaction was stopped and the sample was washed to remove the unconjugated ubiquitin and ligases, polyubiquitin chains were linked to EphA2 by RNF5 (Fig. 1E). EphA2 in vivo ubiquitination was strongly increased by wild-type (WT) RNF5 overexpression but only slightly increased by the RNF5-RING mutated (RM) construct in cells (Fig. 1F). These results indicate that RNF5 induced EphA2 ubiquitination.

Studies have shown that RNF5 regulates autophagy, ER stress and ER-associated degradation [27, 31, 43]. To determine which processes are involved in the regulation of EphA2 by RNF5, we treated the RNF5 WT and KO MEF cells with different stimuli. We found that EphA2 level was greatly increased in RNF5 KO cells compared with RNF5 WT cells, inhibition of proteasome degradation by MG132 treatment increased the EphA2 level in the RNF5 WT cells nearly to that in the RNF5 KO cells, while inhibition of autophagy by chloroquine did not (Fig. 1G), indicating that RNF5 regulates EphA2 degradation through proteasome. Further, the stability of EphA2 was measured in RNF5 WT vs. KO MEFs. After pulse treatment with cycloheximide, the degradation of EphA2 was much slower in the RNF5 KO cells than in the RNF5 WT cells, and the half-lives was extended for ~2 h (from 2.5 to 4.5 h) (Fig. 1H). These results suggest that RNF5 interacts with EphA2 and induces its ubiquitination and degradation.

### RNF5 suppresses EphA2 distribution on the cell surface

**Fig. 2:**
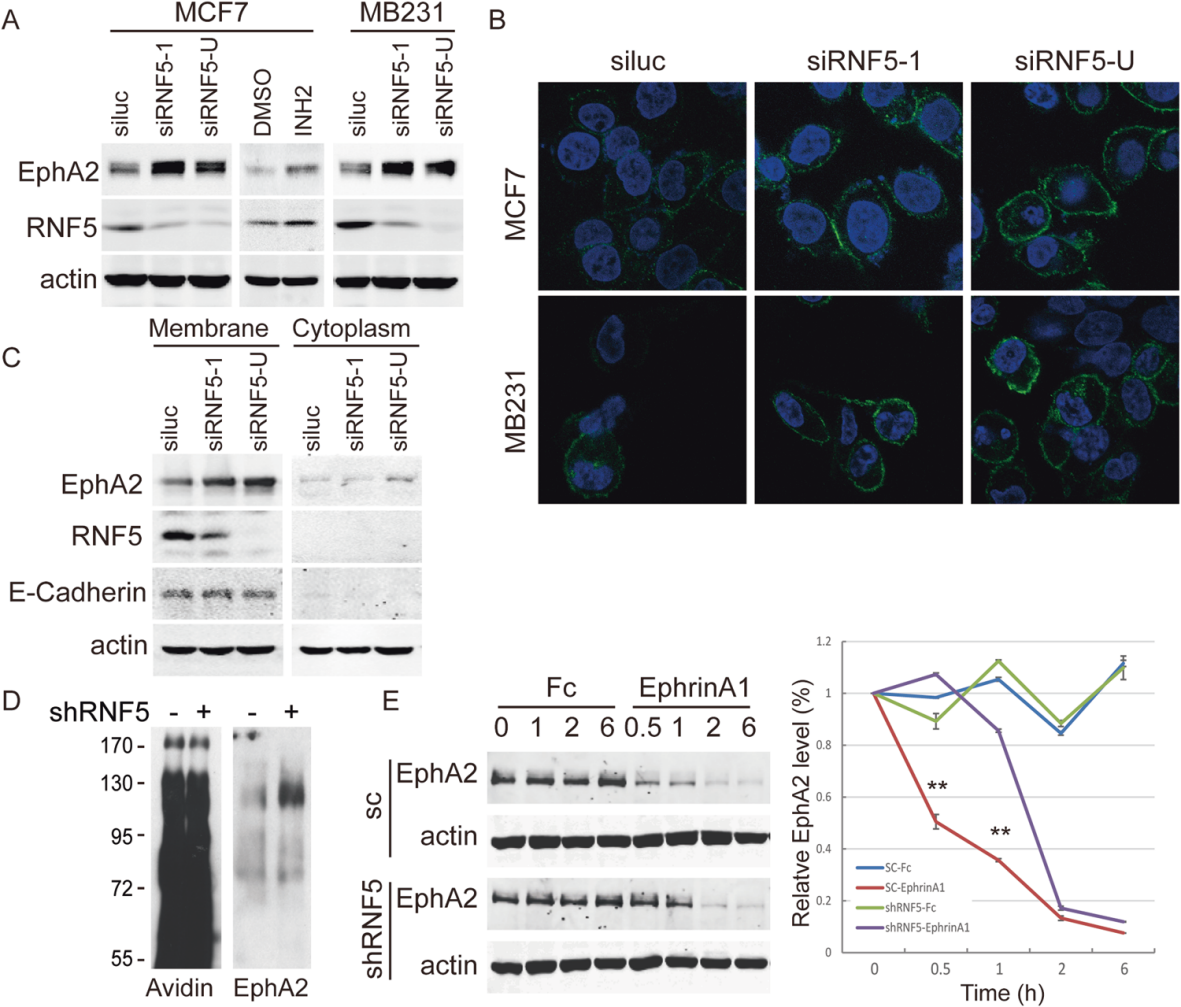
RNF5 inhibition increases EphA2 distribution on the cell surface. **A** The expression of EphA2 in RNF5 wild-type and RNF5 knockdown MCF7 or MDA-MB231 cells was detected by western blotting as indicated. MCF7 cells were left untreated or treated with 10 μM RNF5 inhibitor INH2 for 48 h, and then the cells were collected and detected for the level of EphA2. **B** RNF5 wild-type and RNF5 knockdown MCF7 or MDA-MB231 cells were fixed, permeabilized and analyzed by immunofluorescence staining with an anti-EphA2 antibody; the representative images of EphA2 staining are shown. **C** The membrane and cytoplasm fraction were isolated from RNF5 wild-type and RNF5 knockdown MCF7 cells, and then the membrane and cytosol proteins were extracted and analyzed by western blots as indicated. **D** The cell surface proteins in stable scramble- or shRNF5-transduced MCF7 cells were biotinylated and isolated with streptavidin-affinity gel. The purified proteins were separated by SDS-PAGE and then detected with avidin and anti-EphA2 antibodies by western blots as indicated. **E** Stable scramble- or shRNF5-transduced MCF7 cells were starved overnight in serum-free medium and then treated with 1 μg/ml Fc or Ephrin A1 protein for different times as indicated. The cells were collected, and cell extracts were detected by western blots. The related protein levels were analyzed and shown based on the intensity of grayscale in triplicate experiments. **p < 0.01.

To determine the mechanism by which EphA2 is regulated by RNF5 in breast cancers, RNF5 expression was depleted by shRNA in MCF7 and MDA-MB-231 breast cancer cells, and the level of EphA2 protein in the RNF5 knockdown (KD) cells was found to be significantly higher than that in the RNF5 wild-type (WT) cells (Fig. 2A). Similarly, the RNF5 inhibitor INH2 increased the EphA2 level in MCF7 cells (Fig. 2A). Immunofluorescence staining showed that increased EphA2 level in RNF5 KD MCF7 and MDA-MB-231 cells was observed mainly in the cell surface compartment compared with RNF5 WT cells, while a very low EphA2 signals were observed in the cytoplasm and other intracellular compartments (Fig. 2B). Further, the membrane and cytosol proteins were extracted and the increased EphA2 level was observed in membrane fraction in RNF5 KD cells compared with RNF5 WT cells, while few EphA2 and RNF5 were observed in cytoplasm in both cells (Fig. 2C). These results suggest that RNF5 depletion increases EphA2 level in membrane fraction.

Since EphA2 functions as a cell-surface receptor tyrosine kinase, we further measured whether RNF5 influences EphA2 cell-surface distribution. To further investigate whether the cell-surface distribution of EphA2 may be affected by RNF5 expression, the cell-surface proteins were biotinylated in scramble or shRNF5 transduced cells and isolated by streptavidin-conjugated agarose, increased EphA2 level was observed in affinity-purified biotin-labeled cell-surface proteins from cells transduced with shRNF5 compared with scramble transduction (Fig. 2D). Ligand stimulation of Eph receptors triggers their internalization and degradation, and then scramble- or shRNF5-transduced MCF7 cells were treated with EphrinA1 to induce EphA2 degradation. EphrinA1 treatment induced faster degradation of EphA2 in the scramble-transfected cells than in the shRNF5-transfected cells, while minimal effect on EphA2 was induced by control Fc protein that did not induce EphA2 internalization (Fig. 2E). These results suggest that RNF5 controls the EphA2 cell surface distribution and internalization.

### RNF5 regulates EphA2 phosphorylation and downstream signaling

**Fig. 3:**
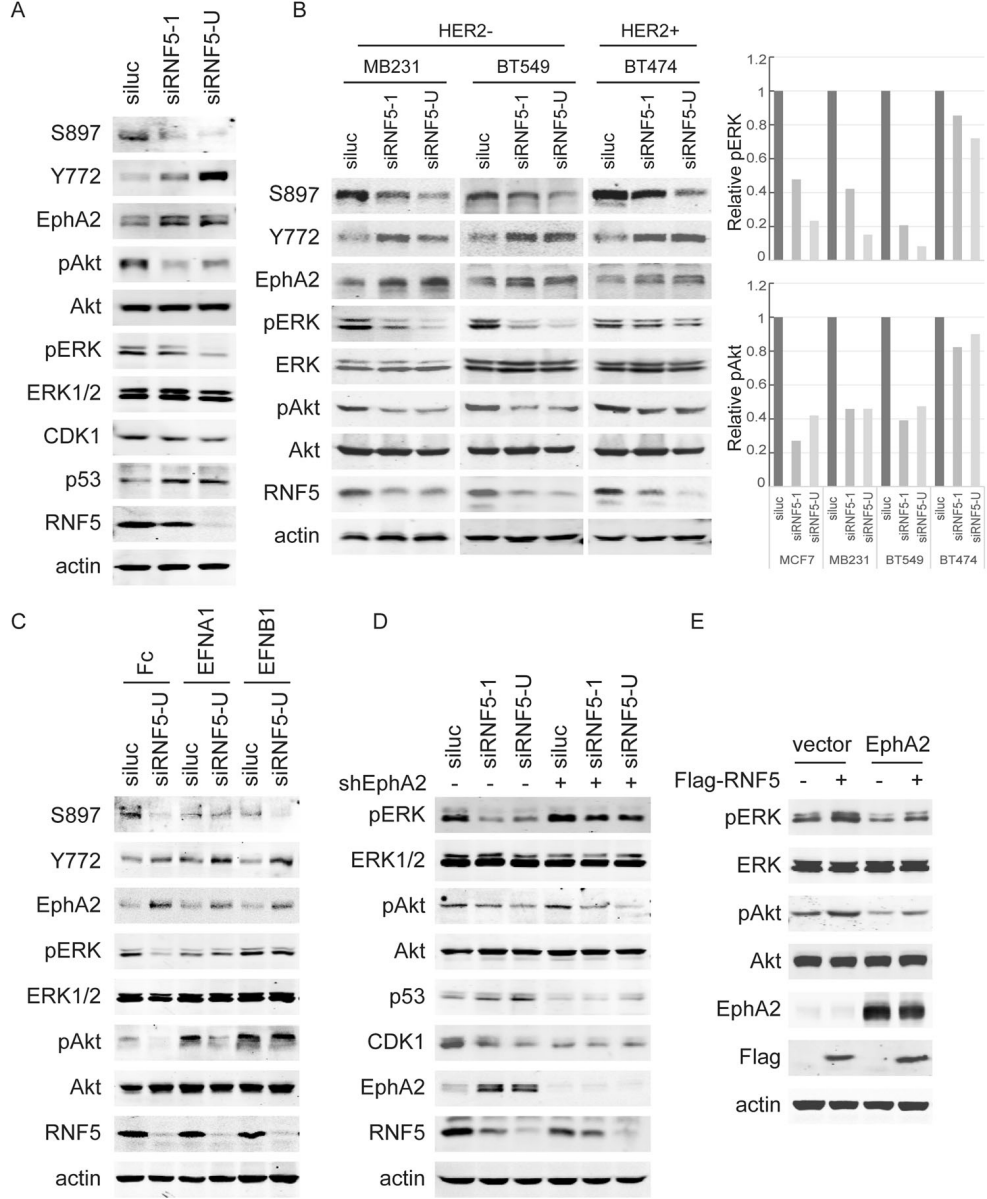
RNF5 depletion increases EphA2 level and alters its phosphorylation to decrease ERK activation in HER2- breast cancer cells. **A** MCF7 cells with stable siLuc or siRNF5 transfection were collected, and the cell extracts were measured by western blots as indicated for EphA2, ERK and Akt phosphorylation and p53 expression. **B** Three lines of breast cancer cells, MDA-MB231, BT549 and BT474, were transduced with scramble shRNA or shRNF5 for 48 h, and then the cell extracts were subjected to western blotting as indicated to evaluate the levels of total and phosphorylated EphA2, ERK and Akt. The related levels of ERK and Akt phosphorylation were analyzed and shown based on the intensity of grayscale. **C** RNF5 WT or RNF5 knockdown MCF7 cells were starved in serum-free medium overnight and then treated with 1 μg/ml Fc, EphrinA1 or EphrinB1 for 20 min. Then, the cells were collected immediately, and the cell extracts were measured by western blots as indicated for EphA2, ERK and Akt phosphorylation. **D** Stable MCF7 cells transduced with siLuc or siRNF5 and non-targeted (NT) or shRNA targeting EphA2 together for 48 h. Then, the cells were collected, and the cell extracts were detected by western blots as indicated for ERK and Akt phosphorylation and p53 expression. **E** MCF7 cells were cotransfected with the empty vector or Flag-RNF5 and the control vector or EphA2-expressing plasmid for 48 h; then, the cells were collected, and the cell extracts were subjected to western blotting as indicated to evaluate ERK and Akt phosphorylation.

To further investigate the effect of RNF5-regulated EphA2 level, we measured EphA2 phosphorylation and several downstream pathways in RNF5 WT vs. KD breast cancer cells. MCF7 exhibits the high level of RNF5 and EphA2 expression, and shRNA-mediated RNF5 knockdown increased EphA2 level and decreased the ERK and Akt phosphorylation in MCF7 cells (Fig. 3A). Similar to ligand-dependent EphA2-Y588 and Y772 phosphorylation and ligand-independent EphA2-S897 phosphorylation [44], the phosphorylation of EphA2 at the S897 site was decreased, while EphA2 phosphorylation at the Y772 site was increased in the RNF5-depleted cells compared to the control cells. These results indicated that RNF5 negatively regulated ERK activation and the balance of EphA2 phosphorylation at Ser897 and Tyr772 sites in tumor cells.

To further investigate whether RNF5 commonly regulates EphA2 phosphorylation and downstream signaling across breast cancers, three other breast cancer cell lines, MDA-MB231, BT549 and BT474, were transfected with scramble or RNF5 shRNA, and EphA2 phosphorylation, as well as ERK and Akt phosphorylation, was quantified. An increase in the EphA2 level was induced by RNF5 shRNA in the three breast cancer cell lines, and the phosphorylation of EphA2 at S897 was decreased while that at Y772 was increased in RNF5 KD cells of all cell lines compared with the corresponding RNF5 WT cells (Fig. 3B, top). These results suggest that RNF5 commonly decreases the EphA2 level and maintains the tumor-promoting status of EphA2 phosphorylation across breast cancer cell lines. The phosphorylation of ERK and Akt was greatly decreased by RNF5 knockdown in HER2-negative MDA-MB231 and BT549 cells compared to the corresponding RNF5 WT cells; however, these phosphorylation was slightly decreased by shRNF5 transduction in HER2-positive BT474 cells (Fig. 3B, middle). Given that EphA2 can interact with HER2 (also named ErbB2) and cooperate with HER2 to activate ERK signaling [45], we concluded that RNF5 inhibition decreases ERK and Akt activation through the increase in the EphA2 level in HER2-negative breast cancer cells.

To further investigate these alterations in EphA2 phosphorylation and downstream signaling in the context of RNF5 depletion, RNF5 WT and KD MCF7 cells were serum-starved overnight and then treated with Fc control, EphrinA1-Fc or EphrinB1-Fc for 20 min. When both RNF5 WT and RNF5 KD MCF7 cells were treated with EphrinA1-Fc or EphrinB1-Fc protein under serum deprivation, the increased EphA2 protein level in RNF5 KD cells compared with RNF5 WT cells were not affected by short-term EphrinA1-Fc or EphrinB1-Fc stimulation, EphA2 S897 phosphorylation in RNF5 WT cells was decreased to the equal level in RNF5 KD cells and EphA2 Y772 phosphorylation was elevated in both RNF5 WT and KD cells by EphrinA1-Fc, while the decreased S897 phosphorylation and increased Y772 phosphorylation were not affected by EphrinB1-Fc treatment (Fig. 3B, top). In addition, the decrease of ERK phosphorylation induced by RNF5 depletion was abolished while the difference of Akt phosphorylation was augmented by EphrinA1-Fc treatment (Fig. 3B, middle), however, EphrinB1-Fc treatment equally induced ERK and Akt phosphorylation regardless RNF5 expression or not, excluding the possibility that RNF5 inhibition regulates ERK and Akt phosphorylation through EphrinB1-related receptor. These results suggest that RNF5 regulates EphrinA1-induced EphA2 phosphorylation and ERK and Akt activation but not affects the signaling of EphrinB1-related receptor.

To further investigate the role of EphA2 in these processes, we depleted EphA2 expression in RNF5 WT and RNF5 KD MCF7 cells and found that EphA2 depletion abolished the difference of ERK phosphorylation in RNF5 KD cells compared with RNF5 WT cells (Fig. 3C), indicating that the decreased ERK activation in the RNF5 shRNA-transduced cells was decided by increased EphA2 level. However, EphA2 depletion did not greatly restore the RNF5-mediated alteration of Akt phosphorylation (Fig. 3C), probably because RNF5 might affect Akt activation mainly through other mechanisms not tightly related to EphA2. Alternatively, EphA2 overexpression decreased and RNF5 overexpression increased ERK and Akt phosphorylation, and RNF5 overexpression greatly restored ERK and Akt phosphorylation in the presence of EphA2 overexpression (Fig. 3D). These results suggest that RNF5 negatively regulates the EphA2 level to promote ERK phosphorylation.

The expression of the cell cycle kinase CDK1 was decreased by RNF5 silencing (Fig. 3A). Furthermore, either RNF5 depletion or EphA2 depletion decreased CDK1 expression in MCF7 cells; however, the CDK1 level was not decreased further by RNF5 silencing in EphA2-depleted MCF7 cells (Fig. 3D), indicating that the expression of both RNF5 and EphA2 is important for CDK1 expression and that RNF5 depletion reduces CDK1 expression through an increase in the EphA2 level, accompanied by unbalanced EphA2 phosphorylation.

The expression of p53 was also negatively regulated by RNF5 in breast cancer cells [24]. We observed that p53 expression was increased in the RNF5-depleted MCF7 cells compared with the control cells (Fig. 3A, D), and the increase of p53 level was attenuated following the decreased EphA2 expression (Fig. 3D). Thus, we conclude that RNF5 inhibition increases p53 expression through the increase of EphA2 level.

### RNF5 decreases adhesion and promotes migration of HER2-negative breast cancer cells by increasing EphA2 level

**Fig. 4:**
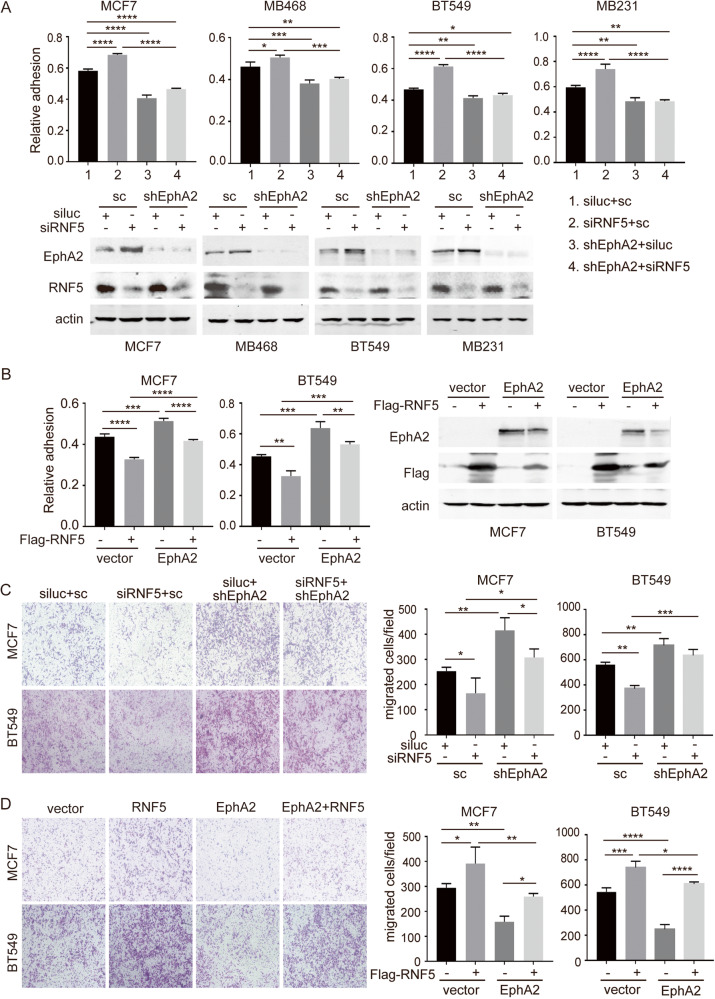
RNF5 regulates cell adhesion and migration through EphA2 in HER2- breast cancer cells. Four lines of HER2− breast cancer cells were cotransfected with siLuc or siRNF5 plus scramble shRNA or shEphA2 (**A**); MCF7 and BT549 cells were cotransfected with the control vector or RNF5-expressing plasmid plus the control vector or EphA2-expressing plasmid (**B**). Forty-eight hours after transfection, cells were harvested by trypsinization and subjected to an adhesion assay. The levels of RNF5 and EphA2 expression were measured by western blotting and are shown as indicated. The relative number of adhered cells was normalized to the total number of cells, and the mean ± SD was calculated from three independent experiments and is shown. *p < 0.05; **p < 0.01, by Student’s *t* test. MCF7 and BT549 cells were cotransfected with shRNAs (**C**) or overexpression plasmids (**D**) as described above. Cells were seeded into the top chambers of transwell plates without a Matrigel coating on the membrane and subjected to a transwell migration assay. The migrated cells on the lower surface of the membrane were stained and visualized by light microscopy, and cell numbers were determined using ImageJ software. The mean ± SD was calculated from three random fields in three independent experiments. *p < 0.05; **p < 0.01, by Student’s *t* test.

To further investigate the effects of RNF5-regulated EphA2 in the tumorigenesis of breast cancer cells, we investigated whether RNF5 affects the adhesion of HER2-negative breast cancer cells. RNF5 depletion resulted in increased adhesion in four lines of HER2-negative breast cancer cells, while EphA2 depletion decreased adhesion and abolished the increase in adhesion in cells with RNF5 depletion (Fig. 4A). In contrast, the adhesion of MCF7 and BT549 cells was decreased by RNF5 overexpression but increased by EphA2 overexpression, and this phenomenon was compromised by combined overexpression of RNF5 and EphA2 (Fig. 4B). These results suggest that RNF5 negatively regulates cell adhesion by targeting EphA2 in HER2-negative breast cancer cells.

The cell proliferation of MCF7 and BT549 cells with or without RNF5 depletion and/or EphA2 depletion were measured, RNF5 depletion alone decreased while EphA2 depletion alone increased cell proliferation in both cells, and double depletion compromised it (Fig. S1A). Similarly, RNF5 overexpression promoted while EphA2 reduced cell proliferation, and the combined overexpression restored it (Fig. S1B). These results suggest that RNF5 positively while EphA2 negatively regulates cell proliferation and their combination compromises the effect. Furthermore, the migration of breast cancer cells was measured in a transwell assay under conditions of RNF5 knockdown and/or EphA2 knockdown. RNF5 knockdown alone decreased while EphA2 knockdown alone increased the migration of MCF7 and BT549 cells, and the decrease in migration resulting from RNF5 knockdown was restored by combined EphA2 knockdown (Fig. 4C). Similarly, RNF5 overexpression positively regulated migration, EphA2 overexpression negatively regulated migration, and combined overexpression compromised these effects in both cell lines (Fig. 4D). These results indicate that the proliferation and migration of HER2-negative breast cancer cells is promoted by RNF5 through a decrease in the EphA2 level. Altogether, these results indicate that RNF5 promotes the tumorigenesis of HER2-negative breast cancer cells by decreasing the EphA2 level.

### Increased EphA2 induced by RNF5 depletion suppresses tumorigenesis

**Fig. 5:**
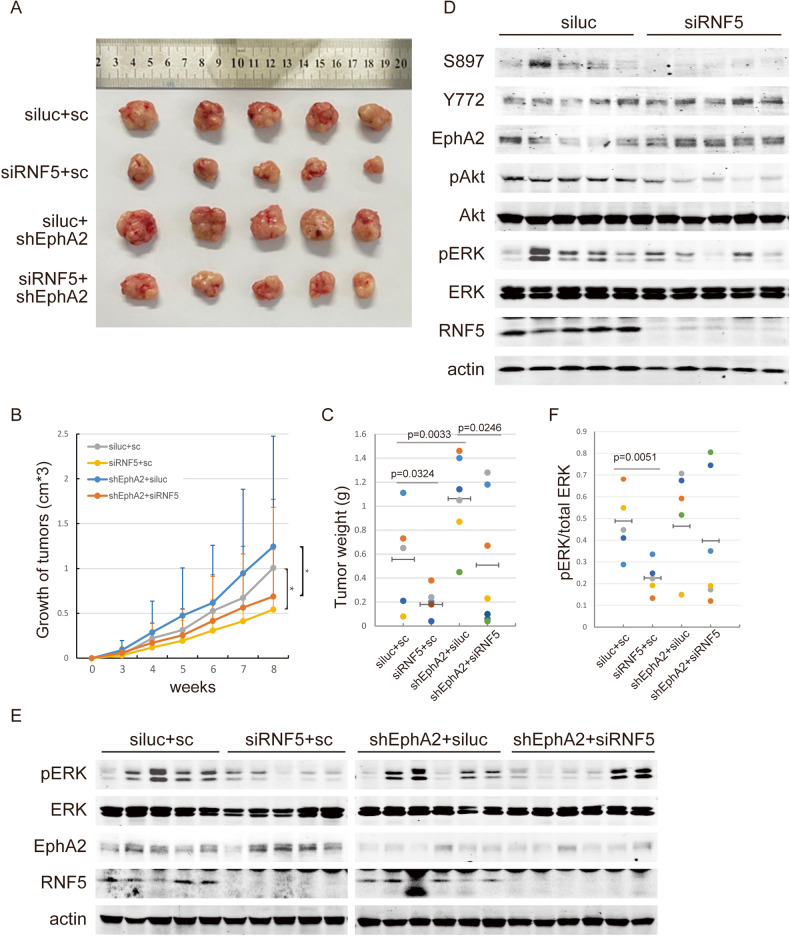
RNF5 depletion suppresses tumor growth through increased EphA2. **A**–**C** Stable siLuc or siRNF5 and scramble (SC) or shEphA2 alone- or together-transduced MCF7 cells were subcutaneously injected into nude mice for 8 weeks. The representative images of tumors were shown (**A**), and the tumor volumes (**B**) and the tumor weight (**C**) were measured, recorded and shown. The *p* value of multiple comparisons are shown. *p < 0.05; **p < 0.01. **D** The whole extracts from siLuc or siRNF5-transduced tumor samples were analyzed by western blots for the expression and phosphorylation of EphA2, ERK and Akt (**C**). **E**, **F** The whole extracts from tumor samples were analyzed by western blots as indicated for ERK phosphorylation (**E**). The related levels of ERK activation were analyzed and shown based on the relative intensity of grayscale of phosphorylated ERK normalized to total ERK level (**F**). The *p* value are calculated by *t*-test and shown.

To further confirm the tumor-suppressive function of EphA2 induced by RNF5 inhibition, we established single or double RNF5- and/or EphA2-knockdown MCF7 cells and injected them into BALB/c nude mice to detect the xenograft tumor growth. The tumor growth was increased by EphA2 depletion while it was decreased by RNF5 depletion, and double depletion exhibited slightly slow growth compared with control tumor growth (Fig. 5A, B). The tumor weights were similarly affected by transduction of EphA2 shRNA alone or RNF5 siRNA alone, and the double transduction abolished their effect and generated the approximately equal tumor weights compared with the control group (Fig. 5C). These results suggest that RNF5 depletion decreased while EphA2 depletion increased MCF7-derived tumor growth and RNF5 attenuated EphA2 tumor-suppressive function. The metastasis of tumor cells in lung in xenograft nude mice was measured using anti-Ki67 staining, however, Ki67-positive cells were affected by neither RNF5 depletion nor by EphA2 depletion (Fig. S2), indicating that RNF5 depletion and EphA2 depletion did not inhibit MCF7-derived tumor cell migration in vivo.

To further explore the mechanism that RNF5 may suppress EphA2 function in tumorigenesis in vivo, the protein samples of xenograft tumors were analyzed. The level of EphA2 was increased in the RNF5-depleted tumors compared with the control tumors (Fig. 5D). The phosphorylation of EphA2, Akt and ERK was further detected and we found that EphA2 S897, Akt and ERK phosphorylation were decreased while EphA2 Y772 phosphorylation was increased in RNF5-depleted tumors compared to control tumors (Fig. 5D). Further, RNF5-depletion decreased ERK activation in tumors while additional EphA2 depletion greatly recovered it (Fig. 5E, F). These results indicate that EphA2 acts as tumor suppressor and RNF5 depletion suppresses the xenograft tumor growth of HER2-negative breast cancer cells through increased level of EphA2 and unbalance of EphA2 phosphorylation. Thus we conclude that RNF5 limits EphA2 level and tumor-suppressive function to facilitate tumor formation of HER2-negative breast cancers.

### High EphA2 level is associated with longer survival in breast cancers

**Fig. 6:**
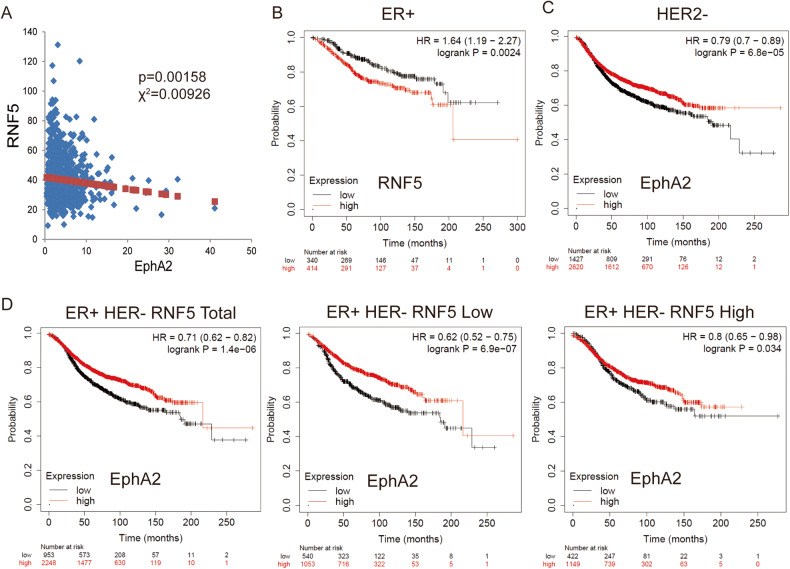
EphA2 acts as tumor suppressor in HER2-negative breast cancers. **A** The expression of RNF5 and EphA2 in breast cancers of TCGA database were analyzed and the correlation of linear regression in breast cancers is shown. The Chi-square test and *p* value are shown. Kaplan‒Meier curves of OS for patients with estrogen receptor-positive breast cancers with low vs. high RNF5 expression (**B**) and of RFS for patients with HER2-negative breast cancers with low vs. high EphA2 expression (**C**). **D** Kaplan–Meier curves comparing the RFS survival of estrogen receptor-positive HER2-negative breast cancers breast cancer patients with tumors expressing low or high EphA2 under total (left), low (middle) and high (right) RNF5 expression level. The data were analyzed by log-rank test, the value of hazard ratio (HR) and log-rank *p* value are shown.

To further investigate the relationship between RNF5 and EphA2, their correlation was further analyzed in human breast cancers. The analysis of RNA-seq levels from TCGA database showed that RNF5 was negatively correlated with EphA2 (coefficients < 0, *p* < 0.01) in breast cancers (Fig. 6A). These results showed that low RNF5 expression is commonly correlated with high EphA2 expression in human breast cancers.

Studies have shown that the lower level of RNF5 expression in breast cancers is related to the longer survival of patients [24]. We investigated the effect of the RNF5-EphA2 level in breast cancers, and then, the survival of breast cancer patients was analyzed with RNF5 and EphA2 levels. Overall survival (OS) of patients with breast cancers showed that a low RNF5 expression level was correlated with better survival in patients with estrogen receptor (ER)-positive breast cancers (Fig. 6B) but with poorer survival in patients with ER-negative breast cancers (Fig. S3A), indicating that estrogen receptor signaling might be required for the tumor-promoting function of RNF5 in breast cancers. Relapse-free survival (RFS) analysis of the total cohort of patients with breast cancer showed that high EphA2 expression was related to better survival in patients with HER2-negative breast cancers (Fig. 6C) but was not correlated with RFS in patients with HER2-positive breast cancers (Fig. S3B), suggesting that the tumor-suppressive function of EphA2 is dependent on the HER2-negative status in patients with breast cancer. Furthermore, the tumor-suppressive function of EphA2 was significantly exhibited in patients with the HER2-negative luminal A subtype (Fig. S3C). Importantly, approximately two-thirds of the breast cancers (3201/4934) in the analysis were ER-positive, HER2-negative, and better survival with high EphA2 expression was similarly observed in this subgroup of patients. The improvement in survival was augmented in the subgroup with low RNF5 expression (Fig. 6D), while it was reduced in the high RNF5 expression subgroup (Fig. 6D). These results suggest that a high EphA2 level is associated with increased survival in HER2-negative breast cancers and that EphA2 suppresses tumorigenesis and the tumor-suppressive function of EphA2 is augmented under low RNF5 expression in ER-positive HER2-negative breast cancers.

Altogether, these results suggest that high EphA2 expression is associated with longer survival of HER2-negative breast cancers with low RNF5 expression, in which leads to that EphA2 acts as a tumor suppressor and improved prognosis under RNF5 inhibition in ER-positive HER2-negative breast cancers.

## Discussion

**Fig. 7:**
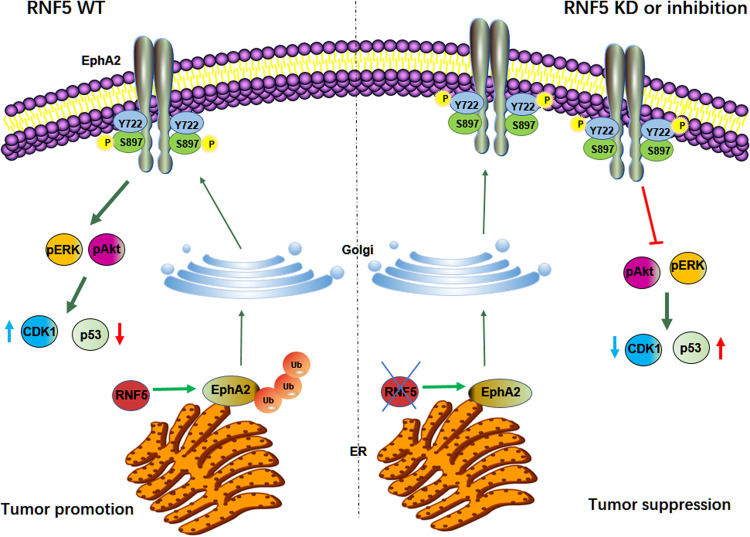
Diagram of RNF5-EphA2 interplay in tumorigenesis. In RNF5 wild-type cells, RNF5 interacts with EphA2 and induces its ubiquitination and degradation, which in turn reduces EphA2 stability and promotes EphA2-S897 phosphorylation to ensure ERK and Akt activation, thus exhibiting a tumor-promoting function. Consequently, RNF5 inhibition or depletion increases the cell surface EphA2 level and alters the balance of EphA2 phosphorylation at S897 and Y772, in turn suppressing ERK and Akt activation and resulting in a tumor-suppressive function.

In present studies, we found that RNF5 interacts with EphA2 and induces its ubiquitination and degradation, to regulate the trafficking and cell-surface distribution. Silencing or inhibition of RNF5 in HER2-negative breast cancer cells decreases ERK activation and EphA2 S897 phosphorylation and increases EphA2 Y772 phosphorylation and p53 expression. As a consequence, increased EphA2 level and altered EphA2 phosphorylation induced by selective RNF5 inhibition decreases the tumorigenesis of HER2-negative breast cancers (Fig. 7). Analysis of patients with breast cancer showed that compared with low EphA2 expression, high EphA2 expression is significantly associated with longer survival times in patients with estrogen receptor-positive HER2-negative breast cancers with low RNF5 expression, who accounts for approximately two-thirds of the breast cancer cohort. Therefore, our studies indicate that RNF5 reduces the stability and cell surface distribution of EphA2 and maintains the balance of its phosphorylation to inhibit its tumor-suppressive function in HER2-negative breast cancers.

Inhibition of EphA2 by inhibitors, conjugated peptides or agonistic antibody is sufficient to induce tumor cell death and inhibit tumor growth [9, 10]; however, several studies have also identified EphA2 as tumor suppressor [13–15]. EphA2 suppresses tumorigenesis under conditions with specific abnormalities such as mutations or genetic dysfunction. Through in vivo shRNA-mediated high-throughput screening, EphA2 was identified as a tumor suppressor in KRas^G12D^-induced human lung cancers with a wild-type TP53 genetic background [13], which carries a mutated K-ras that causes constitutive Akt and ERK activation. Alternatively, the differential phosphorylation of EphA2 determines the effect on tumorigenesis; EphA2-S897 phosphorylation promotes tumorigenesis [20], while EphA2-Y772 phosphorylation decreases transendothelial cell migration of cancer cells [22]. Our results showed that EphA2-S897 phosphorylation is decreased while EphA2-Y772 phosphorylation is increased by RNF5 silencing. EphA2 is accumulated in RNF5-depleted cells in cell surface and easily becomes a dimer or oligomer to induce auto-phosphorylation at tyrosine residues. Although EphA2 interacts with and cooperates with ErbB2 (also named HER2) to activate Ras-MAPK signaling and promote tumorigenesis in HER2-positive breast cancers [45], in HER2-negative breast cancer cells and tumors, the increased EphA2 on the cell surface resulting from RNF5 depletion could be triggered by ligands through cell-cell contact to decrease EphA2-S897 phosphorylation while increasing EphA2-Y772 phosphorylation (Fig. 3C). Consequently, ERK activation was decreased in shRNF5-transduced HER2-negative breast cells and xenograft tumors through the increase in the EphA2 level. And the reduction of activation of ERK-RSK signaling in tumors might further decline EphA2-S897 phosphorylation [20], augmenting the tumor-suppressive function of EphA2. Therefore, our results indicate that RNF5 inhibition could switch EphA2 from tumor-promoting to tumor-suppressive functions by promoting its stability and cell-surface distribution, inducing the unbalance of EphA2 phosphorylation and then decreasing ERK-MAPK activation.

Loss of RNF5 induces innate immunity, antitumor immunity, inflammation and autophagy [25–28, 37], which are highly related to tumor suppression. In fact, the increase in RNF5 expression is associated with poor prognosis and survival of breast cancers [24] and hepatocellular carcinoma [46], and RNF5 degrades PTEN to promote pancreatic tumor growth [38]. In the present study, we confirmed the tumor-suppressive effect of RNF5 inhibition in estrogen receptor-positive HER2-negative breast cancers. RNF5 silencing or inhibition reduced ERK and Akt phosphorylation in HER-negative breast cancer cells that often exhibit constitutive Akt and ERK activation (Fig. 3A, B). As a consequence, low RNF5 expression with high EphA2 expression exhibits tumor-suppressive properties and increased survival in estrogen receptor-positive HER2-negative breast cancers (Fig. 6). However, these results are opposite those of recent studies in which RNF5 inhibits breast cancer progression through the RNF5 association with the glutamine carriers SLC1A5 and SLC38A2 [40] or phosphoglycerate dehydrogenase [47]; unfortunately, these studies used MDA-MB-231 breast cancer cells that is estrogen receptor-negative subtype and carry mutated TP53, and then mutated p53 may not serve as a tumor suppressor but act as a tumor accelerator. Therefore, RNF5 inhibition suppresses tumorigenesis and benefits patients with the wild-type TP53 gene. Analysis of patients with breast cancer showed that the expression status of the estrogen receptor might determine the role of RNF5 in breast cancers and that low RNF5 expression was correlated with better overall survival in patients with estrogen receptor-positive breast cancers (Fig. 6B). High RNF5 levels probably trigger ER stress [31, 35], and induction of ER stress then upregulates estrogen signaling [48] to promote estrogen-dependent breast tumorigenesis. Further analysis of dysfunctions in breast cancers and mining of emerging tumor databases may define the mechanisms, applications and limitations of RNF5-targeted therapy.

As a receptor tyrosine kinase, EphA2 triggers diverse signal transduction pathways and then regulate cell morphology and behaviors. Increased EphA2 induced by RNF5 inhibition reduced ERK activation, while depletion of EphA2 expression recovered ERK activation (Figs. 3D and 5F), suggesting that RNF5 promotes ERK activation at least through EphA2, in turn promoting EphA2 S897 phosphorylation. Furthermore, the expression of tumor-suppressive gene p53 were induced by RNF5 inhibition through increased EphA2 level (Fig. 3D). Although p53 transcriptionally regulates EphA2 expression [49], ERK inhibition causes p53 accumulation in breast cancer cells [50, 51], and high EphA2 levels induced by RNF5 depletion might then promote p53 accumulation by decreasing ERK activation; thus, the reciprocal regulation between p53 accumulation and high EphA2 expression in the context of RNF5 inhibition might amplify p53-related tumor suppression and sensitize tumor cells with wild-type p53 to chemotherapy. In addition, we observed that the expression of cell cycle regulator CDK1 was also decreased by RNF5 inhibition (Fig. 3A), we suppose that RNF5 depletion decreases CDK1 and cell cycle progression through decreasing EphA2 S897 phosphorylation [13, 52]. These data indicate that RNF5 inhibition suppresses several key pathways required for tumorigenesis through increased EphA2 level and the unbalance of EphA2 phosphorylation.

In conclusion, RNF5 suppresses EphA2 by inducing its ubiquitination and degradation; thus, RNF5 inhibition by genetic approaches or inhibitors increases EphA2 level and promotes its tumor-suppressive function by decreasing EphA2 S897 phosphorylation and increasing EphA2 Y772 phosphorylation, decreasing ERK activation and increasing p53 expression, and consequently suppressing tumorigenesis of ER-positive HER2-negative breast cancers. Our studies not only reveal the importance of RNF5 in regulation of EphA2 tumor-suppressive function in breast cancers, but also provide a promising clue to develop novel chemotherapy for ER-positive HER2-negative breast cancers.

## Methods and materials

### Cell lines

Primary RNF5 WT and KO MEFs have the normal growth properties described previously [27] and cultured in DMEM medium supplemented with 10% fetal bovine serum (FBS) and 1% antibiotics (penicillin and streptomycin). HEK293T, MCF-7, MDA-MB-231, BT-549 and BT-474 cell lines were cultured in DMEM medium supplied with 10% FBS and 1% antibiotics after tested for mycoplasma contamination.

### Antibodies, chemicals, plasmids and shRNA

The antibodies, the sequences of shRNA of EphA2 and siRNA of RNF5 are listed in the Supplementary Materials. The RNF5- and shRNF5-expressing plasmids were described previously [27]. EphA2-expressing plasmids were a gift from Dr. Elena Pasquale at Sanford Burnham Prebys Medical Discovery Institute.

### Biotinylation and affinity purification of cell-surface proteins

Cell surface proteins were biotinylated according to the manufacturer’s instructions (EZ-Link™ Sulfo NHS-SS Biotinylation Kit, Thermo Scientific/Pierce, Rockford, lL). Then, the cell extracts were prepared and cell surface proteins were purified by streptavidin-conjugated agarose with elution by 1 μM biotin. The purified biotin-labeled cell-surface proteins were separated by SDS-PAGE and detected by western blots.

### Membrane and cytosol protein extraction

The membrane and cytosol fraction were isolated using homogenization followed by ultracentrifugation. Briefly, the cells were lysed with homogenization in low osmotic solution, and cell extracts were centrifuged at 1000 × *g* for 10 min to remove cell debris and 10,000 × *g* for 10 min to remove mitochondria. The supernatants were ultracentrifuged at 100,000 × *g* for 1.5 h, and then the supernatants containing soluble proteins and the pellets containing all membranes were collected as cytoplasm and membrane fraction, respectively. The membrane proteins were extracted using solution containing 1% Triton X-100 and cytosol proteins were concentrated by Trichloroacetic acid (TCA) precipitation. After protein concentration were determined using BCA protein assay, the membrane and cytosol proteins were analyzed by western blotting analysis.

### Immunoprecipitation and immunoblotting analysis

The cells were transfected with Flag-RNF5 and/or EphA2-expressing plasmids for 48 h, and then, the cells were collected and lysed in the presence of a protease inhibitor cocktail (Roche) and phosphatase inhibitors. For immunoprecipitation, the cell extracts were precleared, incubated with antibodies and then subjected to immunoprecipitation with protein G-agaroses. After a final five washes, the immunoprecipitated complexes were subjected to immunoblotting analysis. For western blots, 40–60 μg protein/lane of the whole cell extract was separated by SDS-PAGE and transferred to NC or PVDF membranes. The membranes were blocked in 5% dry milk and then incubated with primary antibodies and species-matched IRDye® 680- or IRDye® 800- or HRP-labeled secondary antibodies. The images were visualized using the LI-COR Odyssey system or using enhanced chemiluminescence exposed to X-ray film.

### Immunofluorescence

For immunofluorescence, RNF5 WT or knockdown cells cultured in DMEM containing 10% FBS were fixed with 4% formaldehyde in phosphate-buffered saline (PBS) for 15 min, permeabilized with 0.5% Triton X-100 in PBS for 10 min, and then blocked with 1% bovine serum albumin in PBS for 30 min. Then, the cells were incubated with anti-EphA2 primary antibody at 4 °C overnight. After the cells were washed once with PBS and twice with PBS containing 0.1% Triton X-100, they were incubated with Alexa 488-labeled anti-rabbit IgG antibodies (Invitrogen, Carlsbad, CA) for 1 h. The cells were counterstained with DAPI (Sigma, St. Louis, MO) followed by two additional washes. The cells were mounted in antifade agent on glass slides and visualized with a fluorescence microscope.

### Cell adhesion assay

Ninety-six-well plates were pre-coated with 1 μg/ml Fibronectin (100 μl/well) overnight at 4 °C, and then washed three times with 1 × PBS. The cells were seeded into pre-coated 96-well plate at 1 × 10^4^ cells/well and cultured for 8 h at 37 °C. The cell supernatant was discarded and the adherent cells were gently washed with 1 × PBS for three times, with one untreated well as control, and then 100 μl fresh DMEM medium and 2.5 μl MTT (25 μg/ml) was added each well and incubated for additional 2 h at 37 °C. Finally, the cell supernatant was discarded and 100 μl/well DMSO was added to dissolve MTT crystals, and then the absorbance at 490 nm was measured. The relative ability of cell adhesion was normalized to control well and calculated in two independent experiments in triplicate.

### Cell migration assay

The cells were digested and suspended, and then 1 × 10^5^ cells/well in 100 μl serum-free medium were seeded into top chamber with 8 μm-pore filter and inserted to 24-well plate (lower compartment) with 600 μl normal medium containing 10% FBS. After incubated for 24 h at 37 °C the medium was discarded and the chamber was rinsed with PBS and fixed in 4% paraformaldehyde (1 ml/well) for 15–20 min, stained in crystal violet dye solution (1 ml/well) for 15 min, and washed with PBS. Finally, the non-migrating cells on the upper site were gently removed with a cotton swab, and the migrated cells on the lower side were photographed under microscope and the cell number each field were counted using ImageJ software.

### Xenograft tumor growth

One million stable scramble- or shRNA-transduced MCF7 cells were subcutaneously injected into BALB/c nude mice (6–8 week age, female) in random (*n* ≥ 5 each group). Tumor growth was monitored twice per week. When the largest tumors were up to 2 cm in diameter or the animal experiment time arrived 8 weeks, all mice were sacrificed, and the tumors were collected. After the tumor size or weight was determined, the tumors were cut and then fixed with formaldehyde solution or frozen in liquid nitrogen.

### TGCA data mining and analysis

The RNA-seq and survival of breast cancers in The Cancer Genome Atlas (TCGA) were downloaded from https://xenabrowser.net/datapages/. The correlation between RNF5 and EphA2 was performed by linear regression analysis in Excel tools. The Kaplan–Meier survival curves of breast cancers were analyzed by an online survival analysis tool https://kmplot.com/analysis/ with auto select best cutoff as described [53].

### Statistical analysis

The following methods were used for statistical analysis: Student’s *t* test with two-sample unequal variance and two-tailed distribution, *p* values less than 0.05 or 0.01 were defined as significantly. One-way analysis of variance, chi-square test and significance for linear correlation analysis were used. A log-rank (Mantel–Cox) with chi-square test and significance for Kaplan–Meier survival curves were used.

### Supplementary information

Supplementary materials

Original data files

the reproducibility checklist

## Data Availability

The published article and Supplementary files include all datasets generated and analyzed during this study. The additional data are available upon reasonable request.
