# Supplementary material for: Downregulation of EphA2 stability by RNF5 limits its tumor-suppressive function in HER2-negative breast cancers
Source: Cell Death Dis. 2023 Oct 10;14(10):662. doi: 10.1038/s41419-023-06188-y (PMC10564927; doi:10.1038/s41419-023-06188-y)
Supplement: Supplementary file 1 — Supplementary materials [file 41419_2023_6188_MOESM1_ESM.pdf]

# Downregulation of EphA2 stability by RNF5 limits its tumor-suppressive function in HER2-negative breast cancers

Running title: RNF5 suppresses EphA2

Xiaojuan Li[1, †], Fan Wang[2, †] , Lu Huang[2], Mengtian Yang[2] and Ersheng Kuang[2,3,*]

[1] College of Clinical Medicine, Hubei University of Chinese Medicine, Wuhan, Hubei, 430061, China
[2] Zhongshan School of Medicine, Sun Yat-Sen University, Guangzhou, Guangdong, 510080, China
[3] Key Laboratory of Tropical Disease Control (Sun Yat-Sen University), Ministry of Education, Guangzhou, Guangdong, 510080, China

* Correspondence: Ersheng Kuang, E-mail: kuangersh@mail.sysu.edu.cn, Institute of Human Virology, Zhongshan School of Medicine, Sun Yat-sen University North Campus, 74 Zhongshan 2nd Rd, Guangzhou, 510080, China
[†] These authors contributed equally to this work.

**Supplementary methods and materials**

**Table S1. Antibodies**

| Antibody | Catalog number | Company |
|---|---|---|
| p44/42 MAPK | #4695 | CST |
| p-p44/42 MAPK (Thr202/Tyr204) | #4370 | CST |
| Akt | #4691 | CST |
| p-Akt (Ser473) | #4060 | CST |
| EphA2 (8B6) | #12927 | CST |
| EphA2 (D4A2) | #6997 | CST |
| P-EphA2 (Ser897) | #6347 | CST |
| P-EphA2 (Tyr772) | #8244 | CST |
| CDK1 | A2861 | ABclonal |
| p53 | sc-6243 | Santa cruz |
| RNF5 | sc-81716 | Santa cruz |
| Ubiquitin | #3936 | CST |
| HA-Tag | #3724 | CST |
| DYKDDDDK Tag | #2368 | CST |
| Actin | AC004 | ABclonal |

**Purified recombinant proteins:**

Human IgG-Fc

Human EphrinA1-Fc, Sino Biological, 10882-H03H

Human EphrinB1-Fc, Sino Biological, 10894-H03H

**Table S2. Primer pairs**

Primer pairs for shRNAs of EphA2 in pLKO.1 vector (Sigma) with a puromycin resistance gene, and siRNF5 in pSIREN-RetroQ vector (Clontech) with a hygromycin resistance gene.

| | |
|---|---|
| shEphA2-1f | CCGG CGGACAGACATATAGGATATT CTCGAG AATATCCTATATGTCTGTCCG TTTTTG |
| shEphA2-1r | AATTCAAAAA CGGACAGACATATAGGATATT CTCGAG AATATCCTATATGTCTGTCCG |
| shEphA2-2f | CCGG CCATCAAGATGCAGCAGTATA CTCGAG TATACTGCTGCATCTTGATGG TTTTTG |
| shEphA2-2r | AATTCAAAAA CCATCAAGATGCAGCAGTATA CTCGAG TATACTGCTGCATCTTGATGG |
| siRNF5-1f | GATCCG GCGCGACCTTCGAATGTAA TTCAAGAGA TTACATTCGAAGGTCGCGC TTTTTTG |
| siRNF5-1r | AATTCAAAAAA GCGCGACCTTCGAATGTAA TCTCTTGAA TTACATTCGAAGGTCGCGC CG |
| siRNF5-UTR | GATCCG CGGCAAGAGTGTCCAGTAT TTCAAGAGA ATACTGGACACTCTTGCCG TTTTTTG |
| siRNF5-UTR | AATTCAAAAAA CGGCAAGAGTGTCCAGTAT TCTCTTGAA ATACTGGACACTCTTGCCG CG |

**Methods and materials**

Cell proliferation

The cell proliferation were measured by counting cell numbers during cell growth. Briefly, $1\times10^4$/well MCF7 and BT549 cells were seed into 24-well plate, and then cells were digested by trypsinization, the living cells were counted with trypan blue stainin under a light microscopy.

Immunohistochemistry

The immunohistochemistry was performed using a standard procedure. Briefly, the lung samples of xenograft nude mice with MCF7-derived tumors were fixed using paraformaldehyde, embedded with paraffin. The sections were deparaffinized and heated to restore the antigenic epitopes, and then blocked and stained using anti-Ki67 primary antibody and poly-HRP-labelled secondary antibodies. Finally, the sections were detected using DAB substrates, counterstained with hematoxylin solution and visualized with a light microscope.

Li et al. Supplementary Figure S1

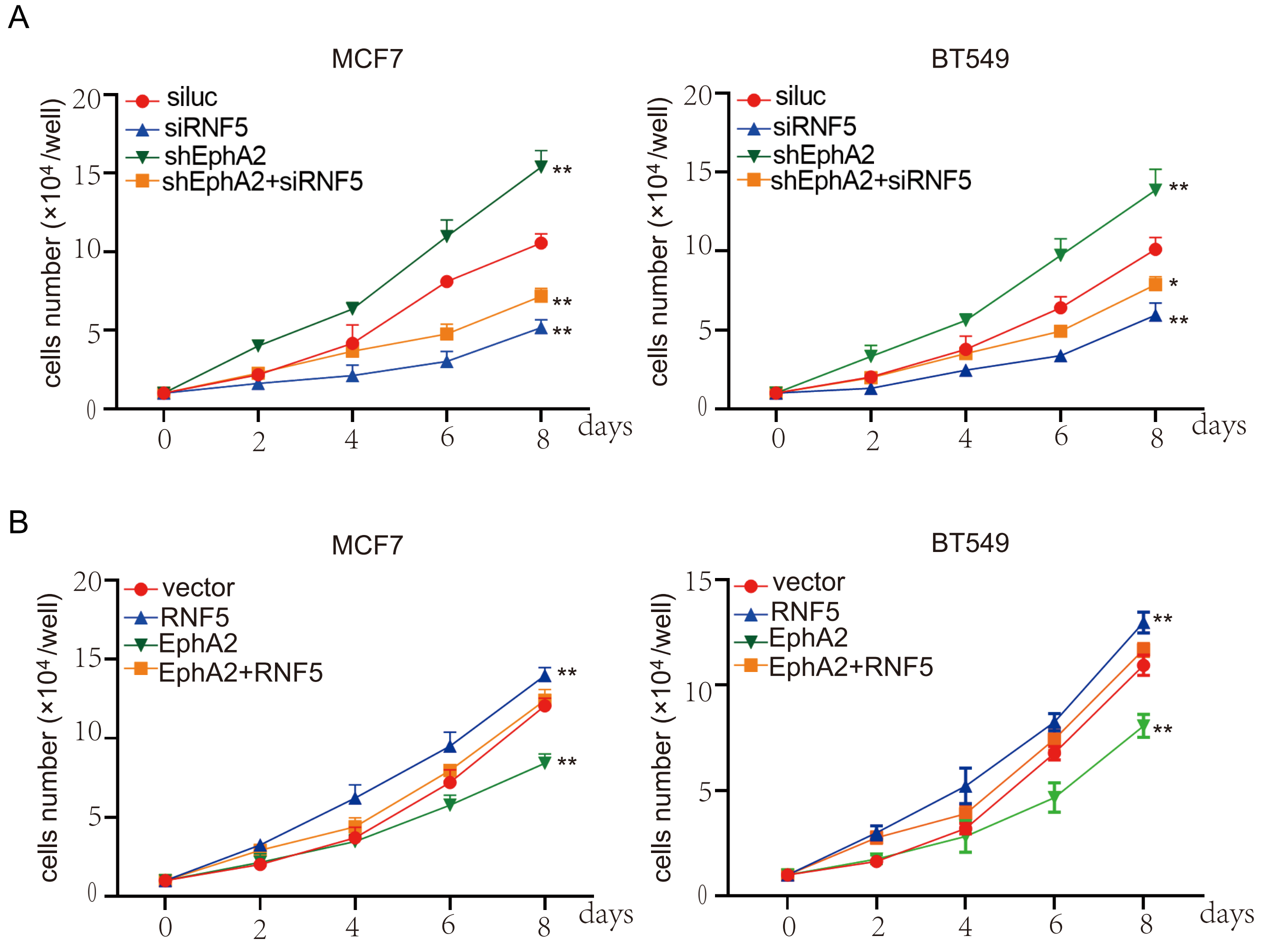


Supplementary Figure S1. The cell proliferation of HER2- breast cancer cells were positively regulated by RNF5 expression while negatively regulated by EphA2 expression

A. Stable siLuc or siRNF5 and scramble or shEphA2 were transduced MCF7 and BT549 cells alone or together, and then cell proliferation were measured at different time points by counting cell numbers.

B. Empty or RNF5 and control or EphA2-expressing plasmids were co-transfected into MCF7 and BT549 cells, the cell proliferation were measured as described above.

The cell numbers were counting in four independent experiments, the means ± SD and p-value of multiple comparisons are shown.. *, $p<0.05$; **, $p<0.01$.

Li et al. Supplementary Figure S2

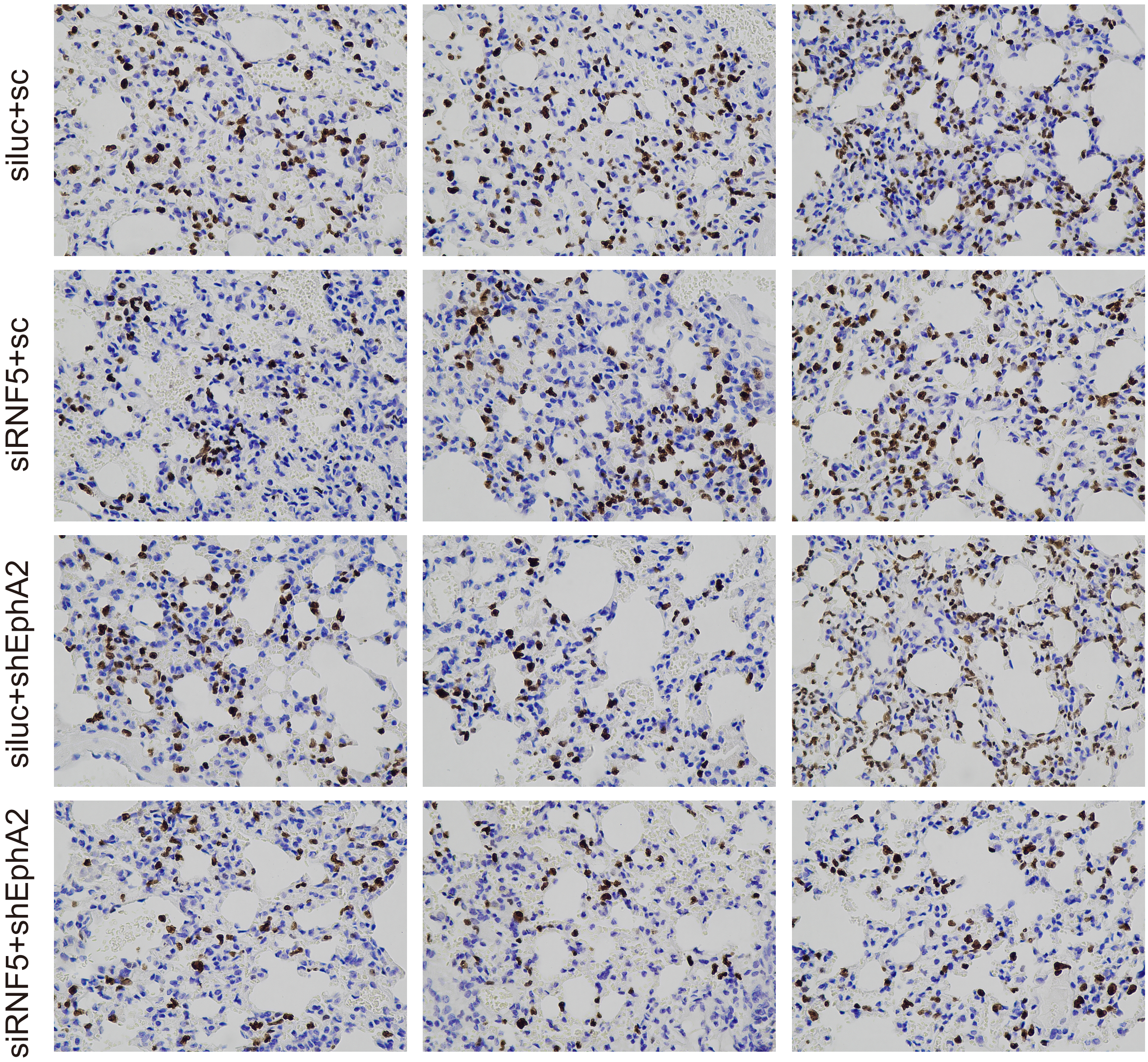


Supplementary Figure S2. RNF5 depletion or EphA2 depletion did not affect the metastasis in vivo

The lung samples of nude mice with MCF7-derived tumors were collected and fixed, and then the metastasis of tumor cells in lung were analysed by immunohistochemistry (IHC) with anti-Ki67 antibody. The representative lung IHC images of three random mice each group were shown (400×).

Li et al. Supplementary Figure S3

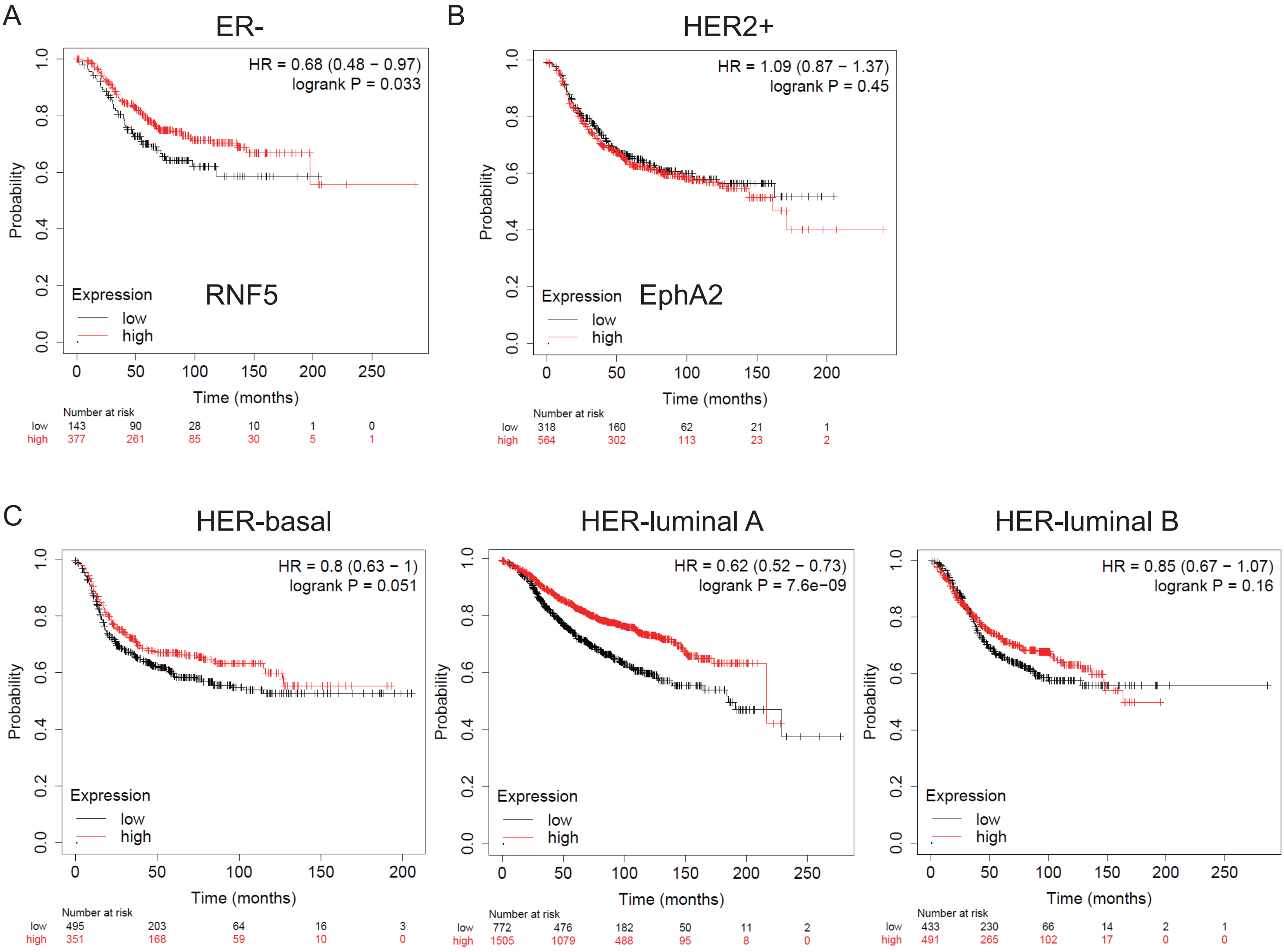


Supplementary Figure S3. Supplementary to Figure 6

A. Kaplan-Meier curves of OS survival of estrogen receptor-negative breast cancers with low vs. high RNF5 expression.

B. Kaplan-Meier curves of RFS survival of HER2-positive breast cancers expressing low vs. high level of EphA2.

C. Kaplan-Meier curves of RFS survival of HER2-negative basal, luminal A or luminal B breast cancers with low vs. high EphA2 level.
