# Supplementary material for: Downregulation of EphA2 stability by RNF5 limits its tumor-suppressive function in HER2-negative breast cancers
Source: Cell Death Dis. 2023 Oct 10;14(10):662. doi: 10.1038/s41419-023-06188-y (PMC10564927; doi:10.1038/s41419-023-06188-y)

Li et al. Supplementary Figure S4A

Uncropped immunoblots related to Figure 1A, 1B,1C, 1D and 1E

Fig 1A

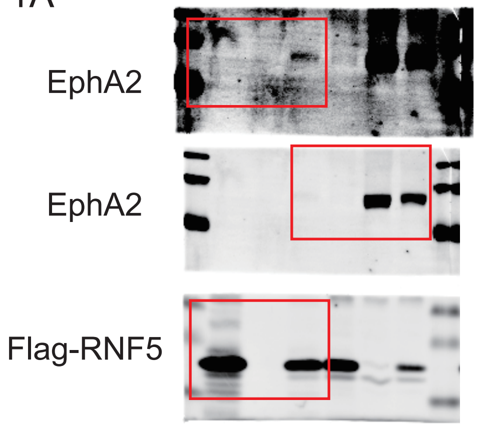

Fig 1B

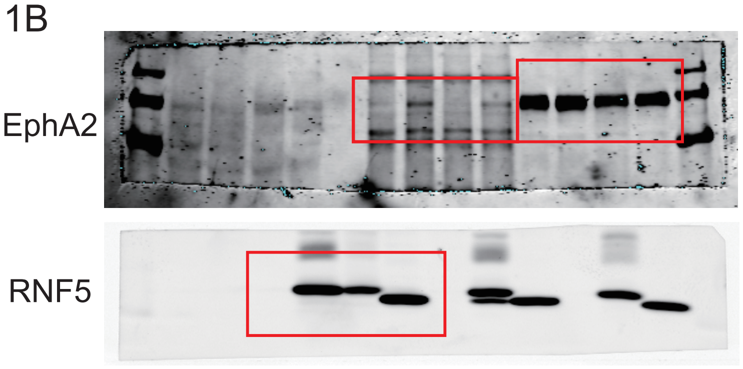

Fig 1C

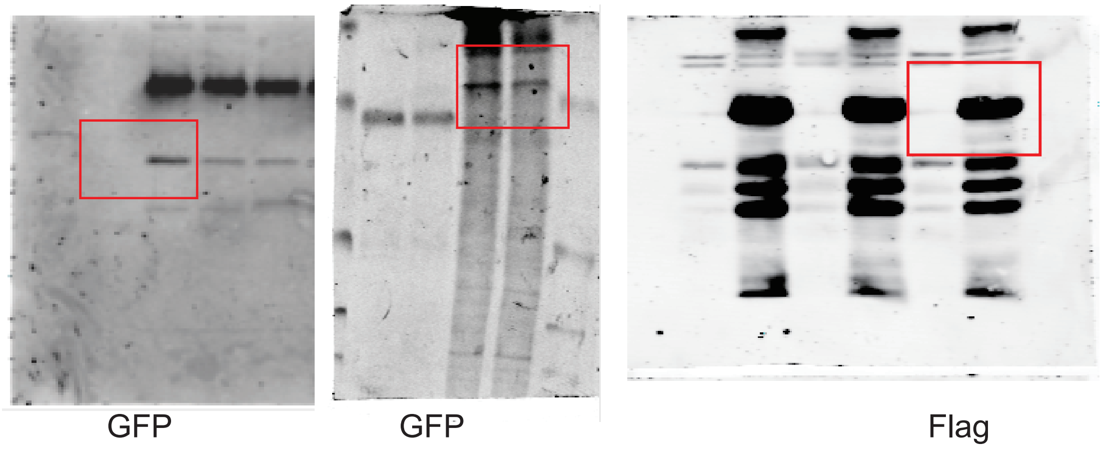

Fig 1D

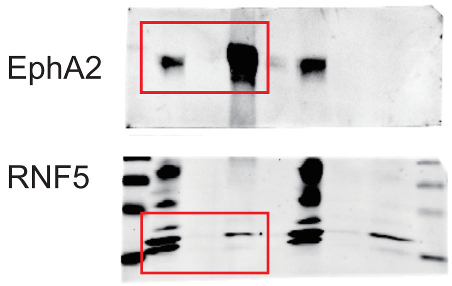

Fig 1E

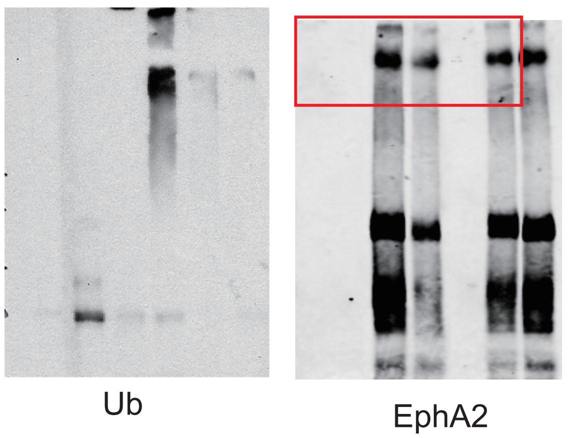

Li et al. Supplementary Figure S4B

Uncropped immunoblots related to Figure 1F, 1G and 1H

Fig 1F

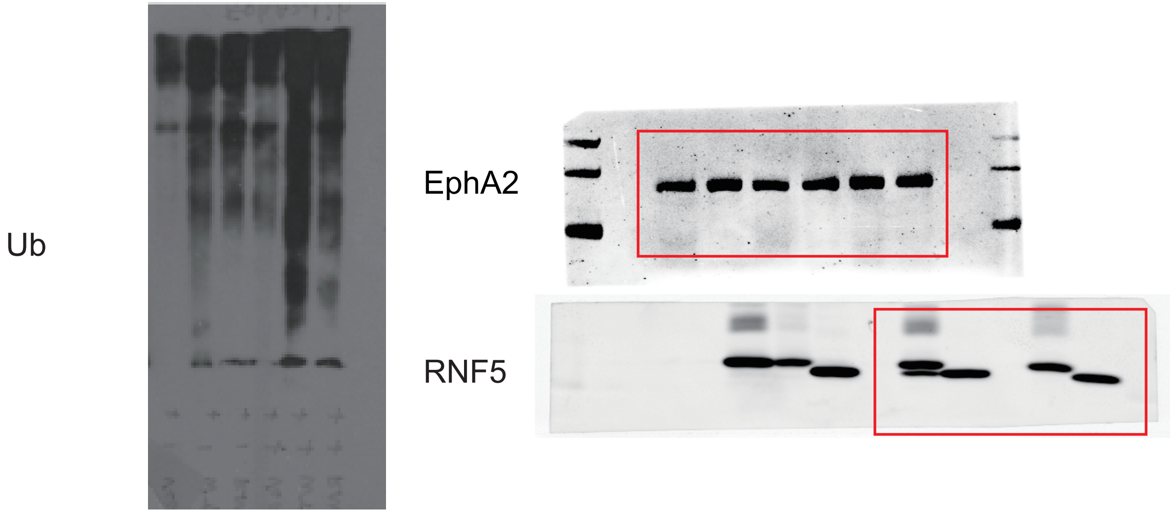

Fig 1G

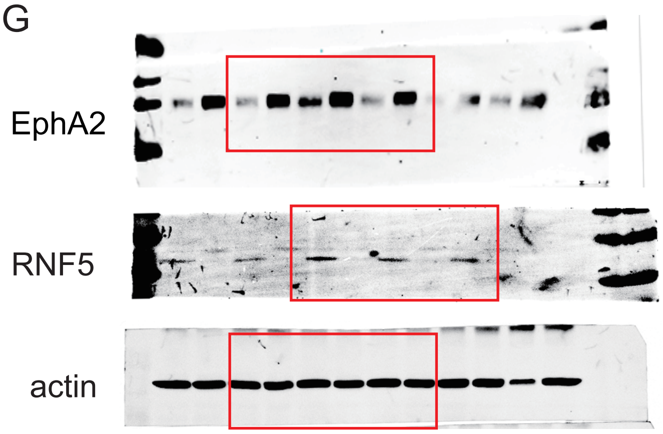

Fig 1H

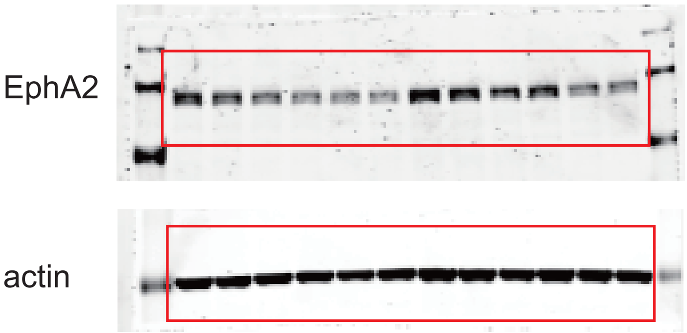

Li et al. Supplementary Figure S4C

Uncropped immunoblots related to Figure 2A, 2C, 2D and 2E

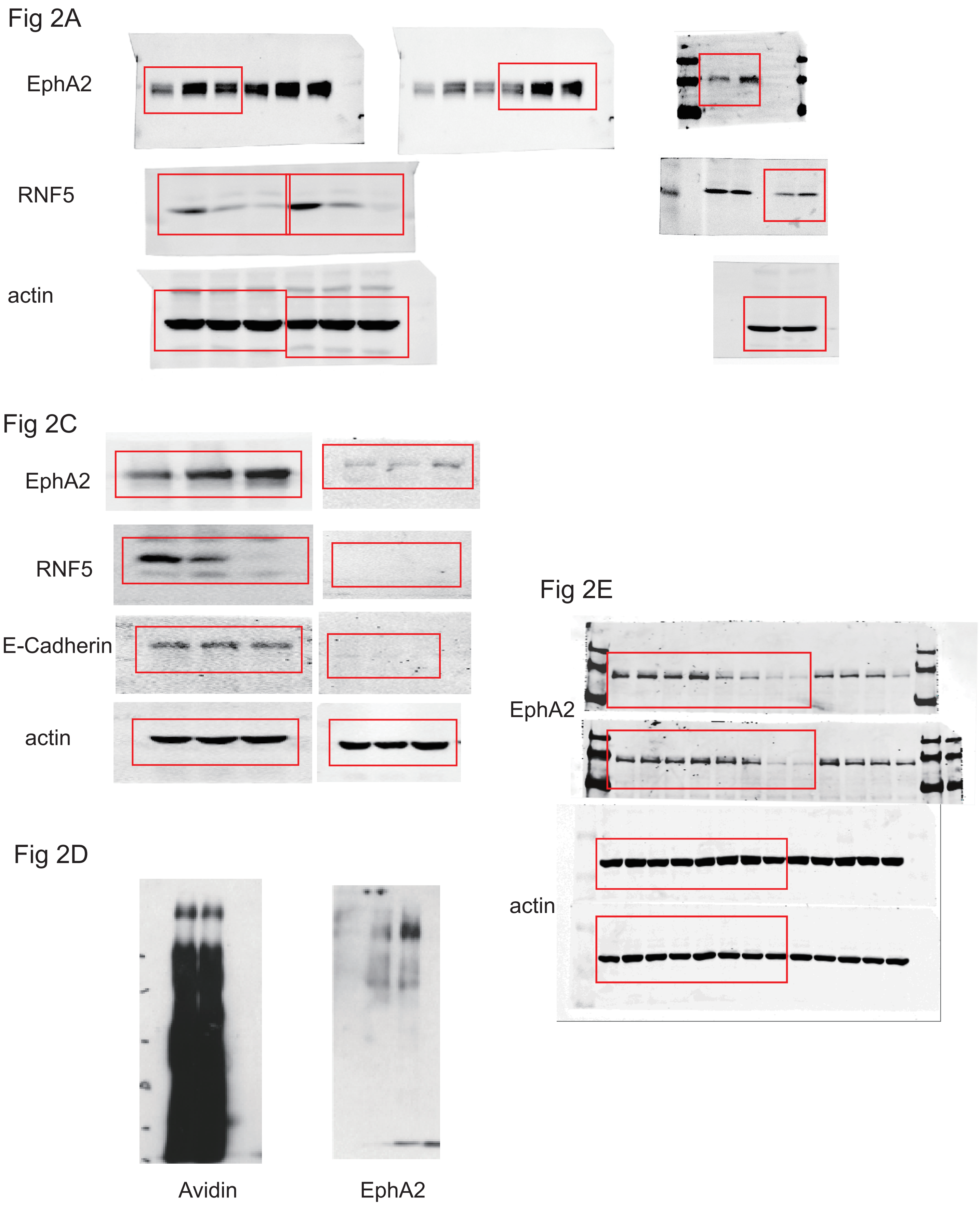

Li et al. Supplementary Figure S4D

Uncropped immunoblots related to Figure 3A and 3B

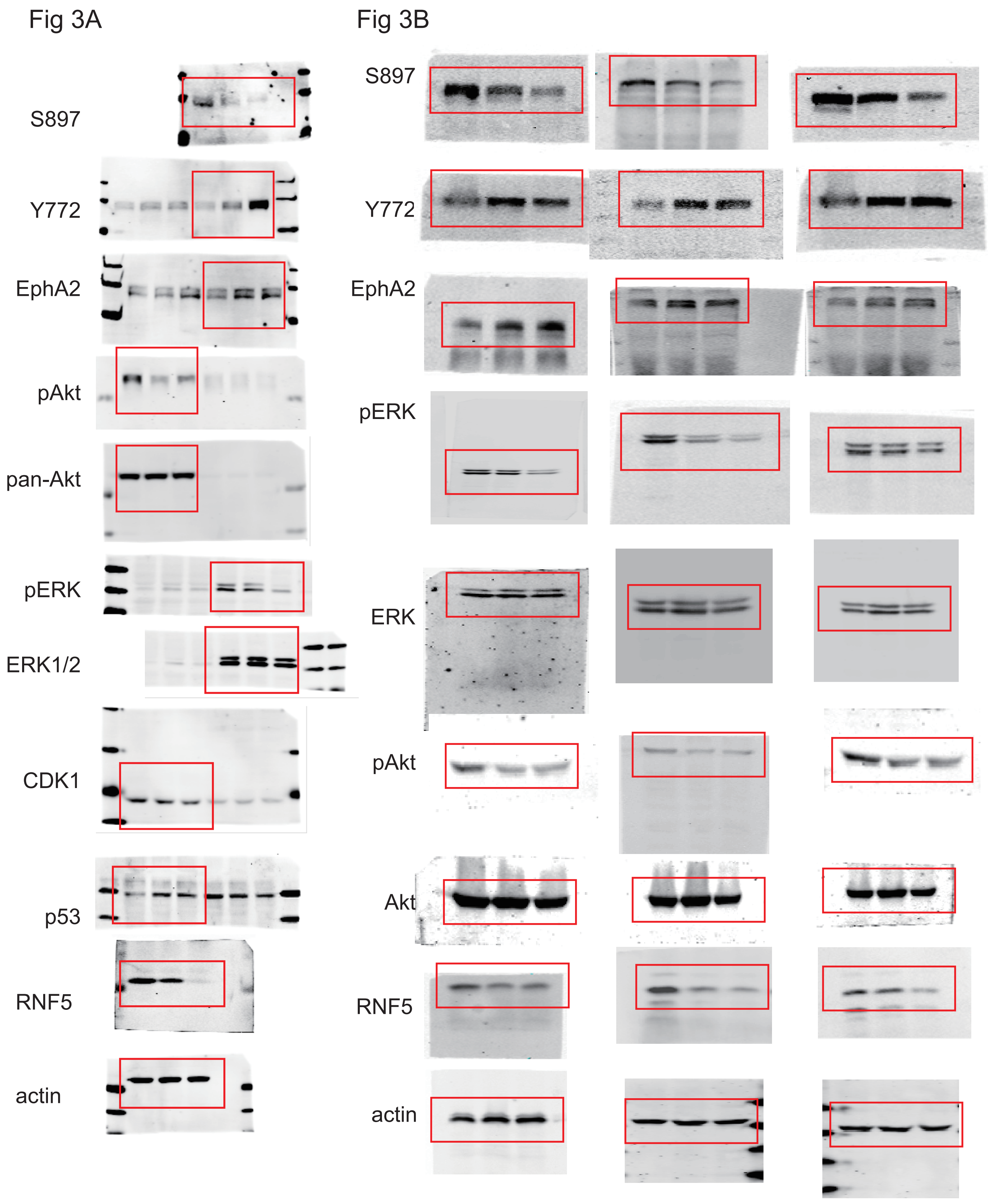

Li et al. Supplementary Figure S4E
Uncropped immunoblots related to Figure 3C, 3D and 3E

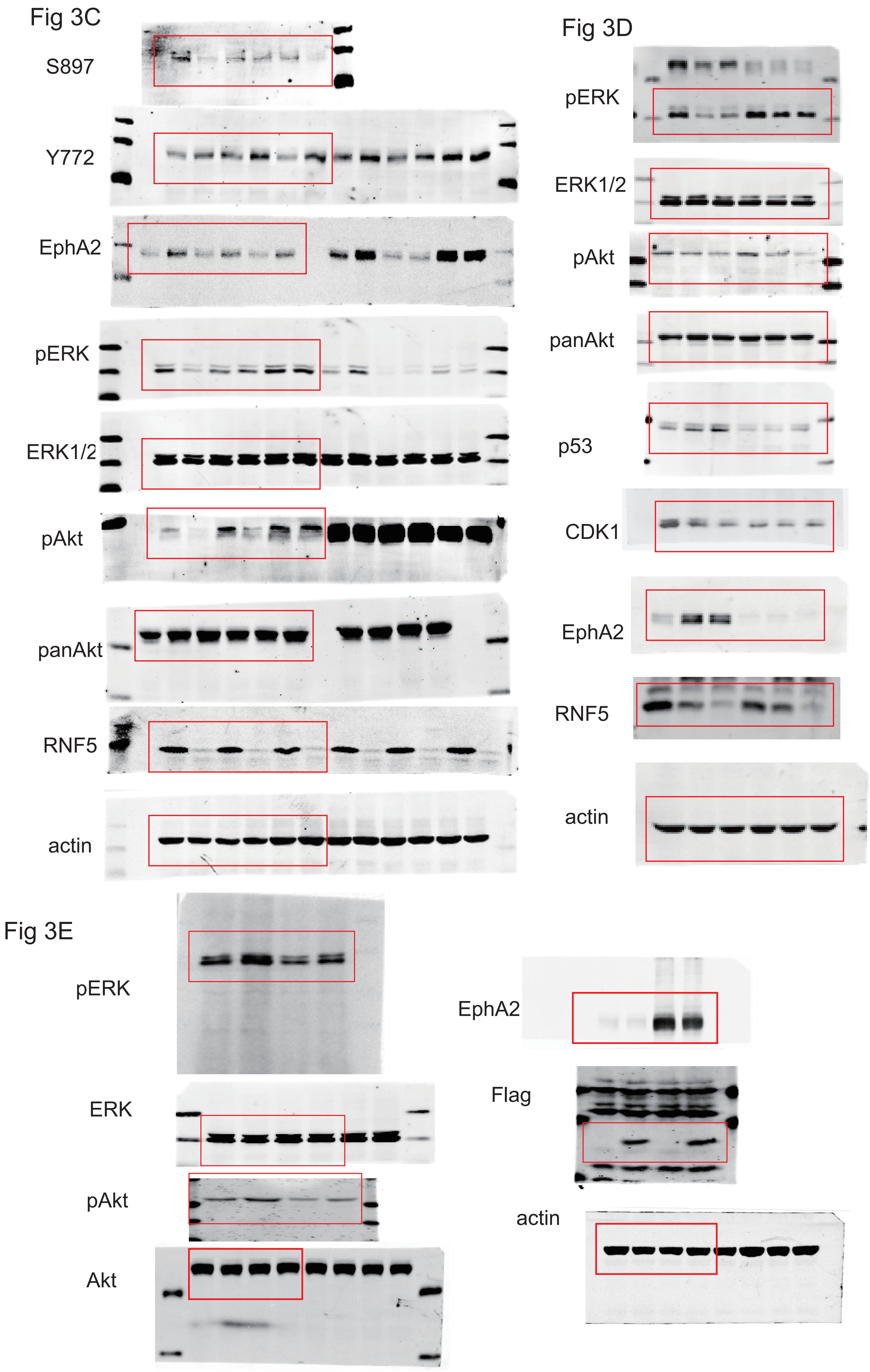

Li et al. Supplementary Figure S4F

Uncropped immunoblots related to Figure 4A and 4B

Figure 4A

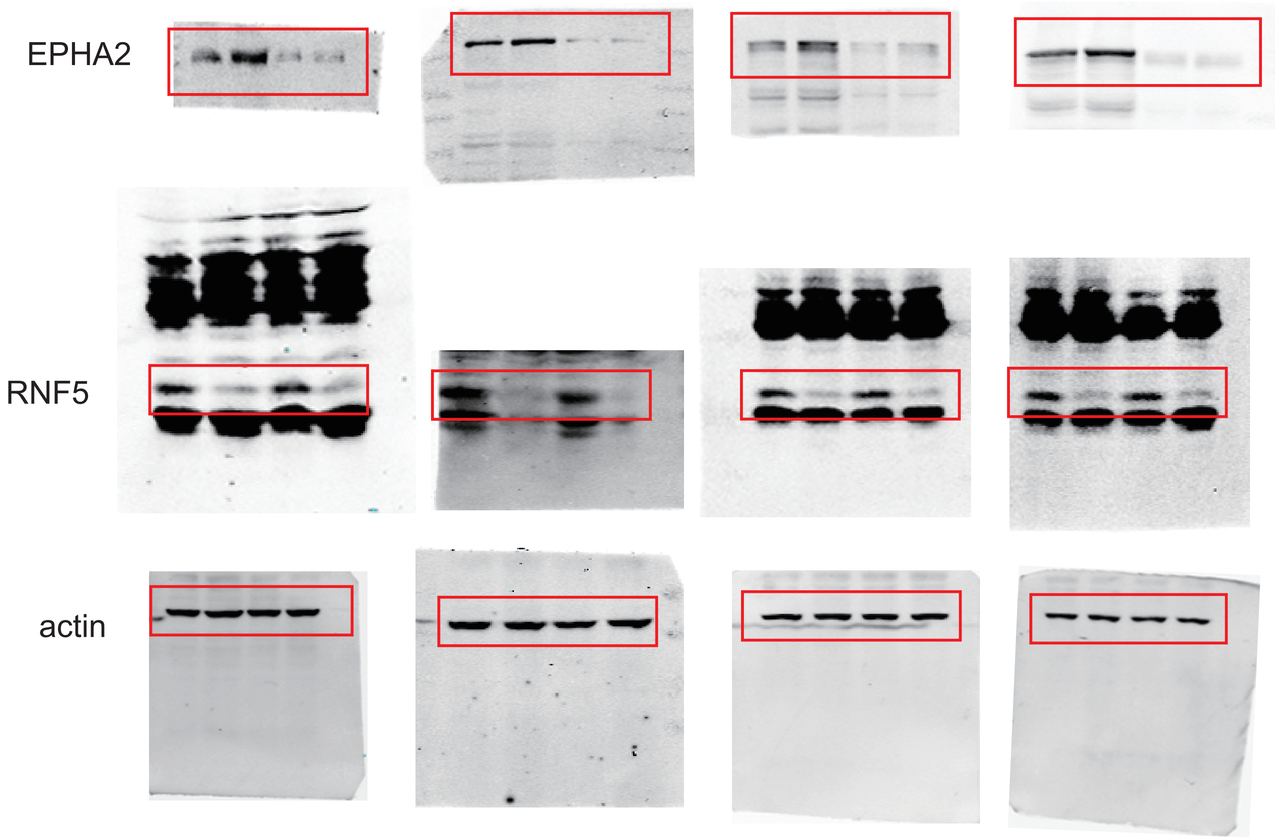

Figure 4B

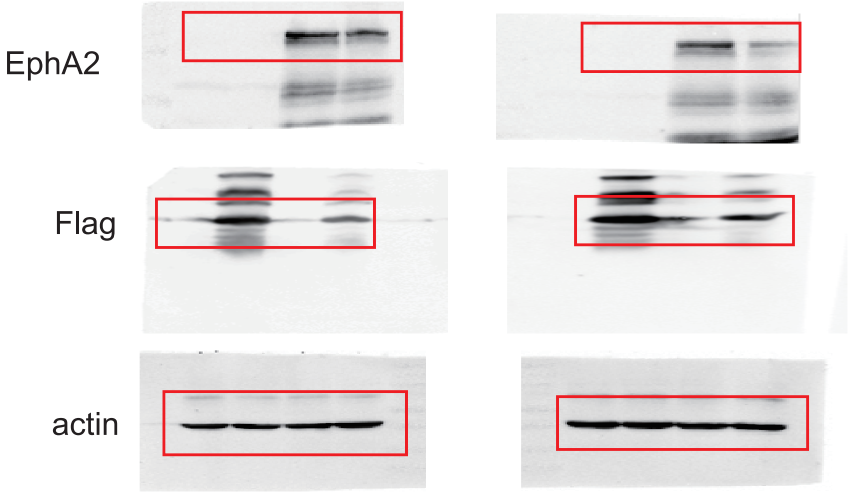

Li et al. Supplementary Figure S4G

Uncropped immunoblots related to Figure 5D and 5E

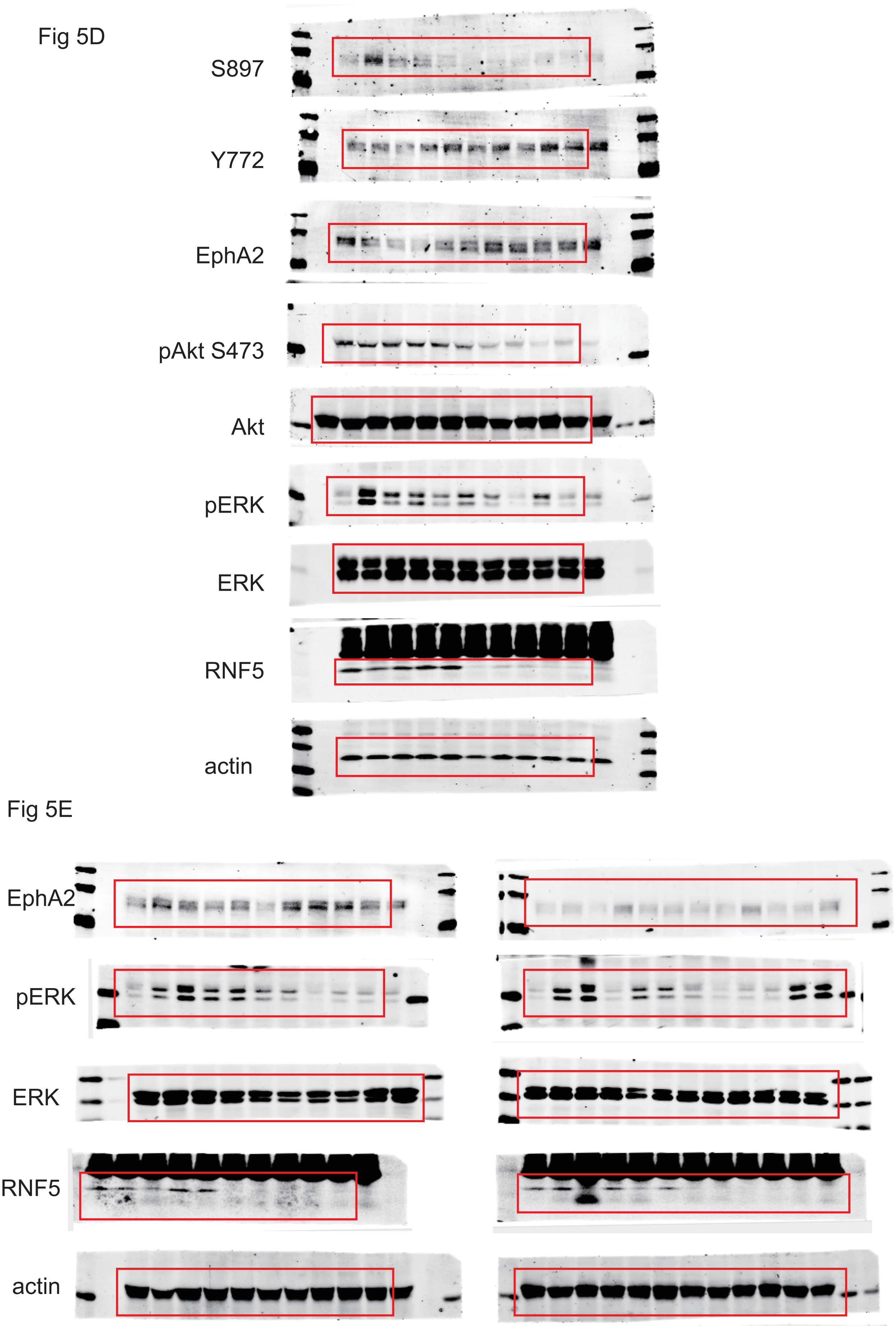

Supplement: Supplementary file 2 — Original data files [file 41419_2023_6188_MOESM2_ESM.pdf]
